# *In vivo* spontaneous activity and coital-evoked inhibition of mouse accessory olfactory bulb output neurons

**DOI:** 10.1016/j.isci.2023.107545

**Published:** 2023-08-07

**Authors:** Paolo Lorenzon, Kamil Antos, Anushree Tripathi, Viktoria Vedin, Anna Berghard, Paolo Medini

**Affiliations:** 1 Department of Integrative Medical Biology, Umeå University, SE90187 Umeå, Sweden; 2 Department of Molecular Biology, Umeå University, SE90187 Umeå, Sweden

**Keywords:** Neuroscience, Behavioral neuroscience, Sensory neuroscience, Cell biology

## Abstract

Little is known about estrous effects on brain microcircuits. We examined the accessory olfactory bulb (AOB) *in vivo*, in anesthetized naturally cycling females, as model microcircuit receiving coital somatosensory information. Whole-cell recordings demonstrate that output neurons are relatively hyperpolarized in estrus and unexpectedly fire high frequency bursts of action potentials. To mimic coitus, a calibrated artificial vagino-cervical stimulation (aVCS) protocol was devised. aVCS evoked stimulus-locked local field responses in the interneuron layer independent of estrous stage. The response is sensitive to α1-adrenergic receptor blockade, as expected since aVCS increases norepinephrine release in AOB. Intriguingly, only in estrus does aVCS inhibit AOB spike output. Estrus-specific output reduction coincides with prolonged aVCS activation of inhibitory interneurons. Accordingly, in estrus the AOB microcircuit sets the stage for coital stimulation to inhibit the output neurons, possibly via high frequency bursting-dependent enhancement of reciprocal synapse efficacy between inter- and output neurons.

## Highlights

• AOB output neurons are more hyperpolarized and fire high frequency bursts in estrus
• Calibrated artificial vagino-cervical stimulation inhibits spiking only in estrus
• α1-adrenergic receptor activation of reciprocal synapses is key to spike inhibition

## Graphical abstract

**Figure:**
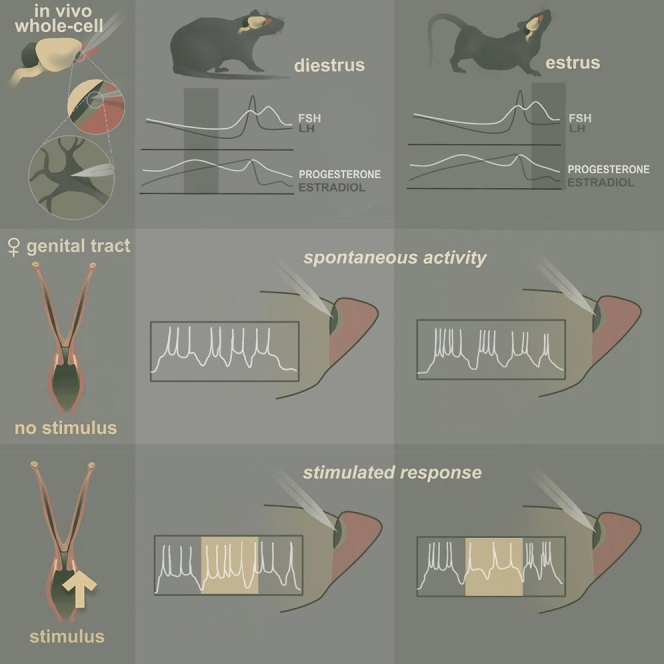

## Introduction

Given the fundamental importance of coitus for life itself, it is surprising that the neural regulation thereof is an understudied area in mammals, while e.g., genes governing copulatory behavior in *Drosophila* are known to a relatively great level of detail (example in Shao et al.^1^). Coitus is only part of the complex behavioral mating sequence that starts with social interaction of the couple. In the mouse, the neuroendocrinological state of the female is pivotal for the mating to occur. During the hours when the female brain can be said to be in an estrous state, she may accept a male for mating.^2^ Outside of estrus the female engages in other kinds of social behavior. The estrous cyclicity impacts on brain function by e.g., fine structure changes of dendritic spines,^3^ modulation of synaptic function,^4^ altered sensory responsiveness,^5^ and microcircuit plasticity.^6^^,^^7^ There conceivably are many brain regions, which receive somatosensory information that is generated by vagino-cervical stimulation during coitus (e.g., Lenshow et al.^8^), but overall knowledge is sketchy and particularly at the microcircuit level. One part of the brain that is classically known to show plasticity upon mating is the female accessory olfactory bulb (AOB) where a physiological recognition memory for the mating male is formed.^9^^,^^10^ An individual male is recognized by his specific blend of odorous compounds and the non-volatiles stimulate neurons in the vomeronasal organ (VNO) rostral in the nose, which in turn leads to activation of the AOB. These compounds often are termed pheromones, but as odors with such qualities also are recognized by the main olfactory system, we herein use the term VNO-pheromones.^11^^,^^12^^,^^13^ If another male takes over the territory within a few days after the female has mated, then this AOB stimulation by the new male’s VNO-pheromones will lead to suppression of prolactin release from the pituitary. If so, the female will not implant the embryos from the mating male, but instead re-enter the estrous cycle.

Several studies taken together suggest that the mating signal necessary to form this type of memory in the AOB, is mediated by the noradrenergic projection from the locus coeruleus.^10^^,^^11^^,^^14^ The electrophysiological effects of norepinephrine (*also* noradrenaline) on the AOB microcircuit have been studied in brain slices and *ex vivo*, but it is not straightforward to synthesize the available data into one model and most results are from peripubertal mice of mixed sexes. Norepinephrine has been suggested to stimulate as well as inhibit AOB mitral-tufted output neurons, either directly or indirectly via granule cells (GC).^15^^,^^16^^,^^17^^,^^18^ Moreover, there are heterogeneities in, and age-dependence of, the neuronal responses.^15^^,^^16^^,^^17^ Thus, several cell physiological properties appear influenced by norepinephrine, but which mechanisms that predominate *in vivo* in the adult female is not known and differences in electrophysiological responses between *in vitro* and *in vivo* are expected.^19^^,^^20^ The AOB microcircuit may be relatively simple, still the specialized reciprocal dendro-dendritic synapses between the GABAergic GC interneurons and glutamatergic mitral-tufted output neurons need to be considered in any model put forward. In the same neuron, the reciprocal synapse’s presynaptic active zone with synaptic vesicle release capacity is directly juxtaposed to membrane with postsynaptic functions. In AOB the reciprocal synapses are found on primary dendrites of the output neurons, while these predominantly are on lateral dendrites in the more studied main olfactory bulb,^21^ suggesting functional differences in their role in modulation of input signals.

We here demonstrate a methodological approach to compare the effects of calibrated artificial vagino-cervical stimulation (aVCS) on sub- and suprathreshold activities of neurons in anesthetized, spontaneously cycling, female mice in estrus, and diestrus. A reductionistic experimental approach is necessary to specifically study the coital somatosensory component of the otherwise complex multisensory integration occurring during the mating process.

The area of choice is the AOB, for which we are first to report intracellular recordings of *in vivo* responses of mitral-tufted output neurons in response to the coital-like stimulation, in estrus versus diestrus. We show that in naturally cycling females aVCS inhibits the spiking of AOB output neurons only during estrus. In addition, our approach reveals differences in the intrinsic excitability and action potential firing pattern in estrus versus diestrus and we propose a causal link between these two observed phenomena.

## Results

### Artificial vagino-cervical stimulation recruits locus coeruleus and evokes responses in AOB

First a functional anatomy study was done to test whether aVCS activated locus coeruleus, and if so, to what extent. The GC layer of AOB harbors inhibitory GCs, innervated by locus coeruleus fibers as shown by anterograde labeling in the rat*.*^22^ Locus coeruleus releases norepinephrine in AOB upon mating, or aVCS, in the mouse.^10^^,^^14^ *In vitro* and *ex vivo* studies suggest different potential outcomes of norepinephrine in AOB, largely mediated by α1-adrenergic receptors^15^^,^^16^^,^^17^^,^^18^ that are expressed by GCs (subtype α1A; a few cells in the mitral-tufted cell layer express α1D^23^^,^^24^).

**Figure 2:**
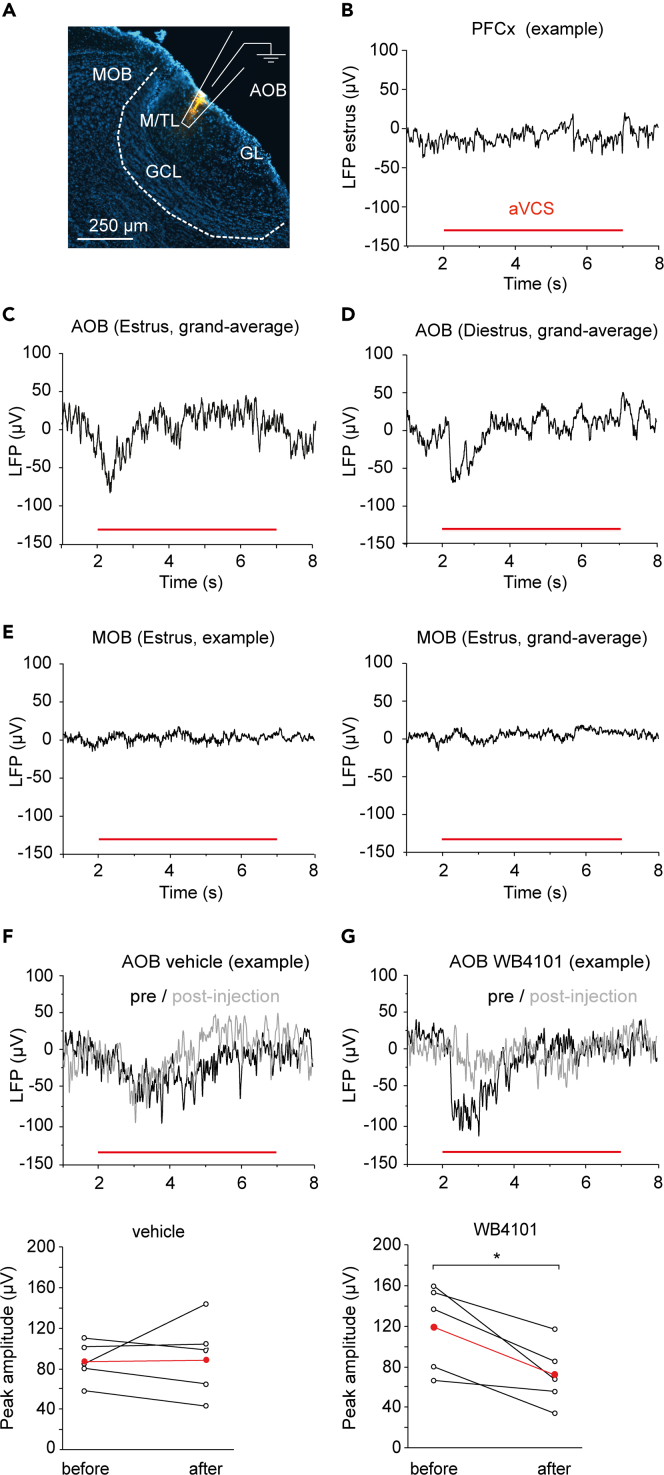
Artificial vagino-cervical stimulation gives rise to local field potential responses of adrenergic origin selectively in the accessory olfactory bulb (A) Shows a sagittal AOB section with a DiI-labeled electrode path (orange) targeted to the mitral-tufted layer (M/TL; DAPI stained nuclei in blue). The terminals of vomeronasal sensory axons synapse on the only output neurons i.e., M/T neurons, in the glomerular layer (GL). M/T cell bodies are in the M/TL and receive input also from inhibitory GC interneurons that form reciprocal synapses on the dendrites of M/T neurons, in the M/TL. The cell bodies of GCs are in the GC layer (GCL). Part of the main olfactory bulb (MOB) is visible. Red lines in B–G indicate aVCS period. (B) Example LFP recording showing lack of aVCS response when the electrode was in prefrontal cortex (PFCx). (C and D) Grand average LFP responses in M/TL evoked by aVCS in estrus (N = 7) and in diestrus (N = 5). Note the transient and stimulus-locked nature of the response. (E) Lack of aVCS-evoked LFP response in the main olfactory bulb (left: example, right: grand average; N = 5). (F) Upper panel shows an example of aVCS-EPs evoked in M/TL before (black) and after (gray) intra-parenchymal injection of vehicle in AOB. Lower panel shows quantification of LFP peak amplitudes recorded before and after vehicle injection (N = 5 estrus females). (G) Upper panel shows an example of aVCS-EPs evoked in M/TL before (in black) and after (in gray) intra-parenchymal injection of the α1-adrenergic receptor blocker WB4101. Lower panel shows quantification of the LFP peak amplitudes in response to aVCS recorded before and after (30 min) WB4101 injection (N = 5 estrus females; paired t-test, ∗p < 0.05). The mean values are shown in red in the quantification panel for F and G.

**Figure 1:**
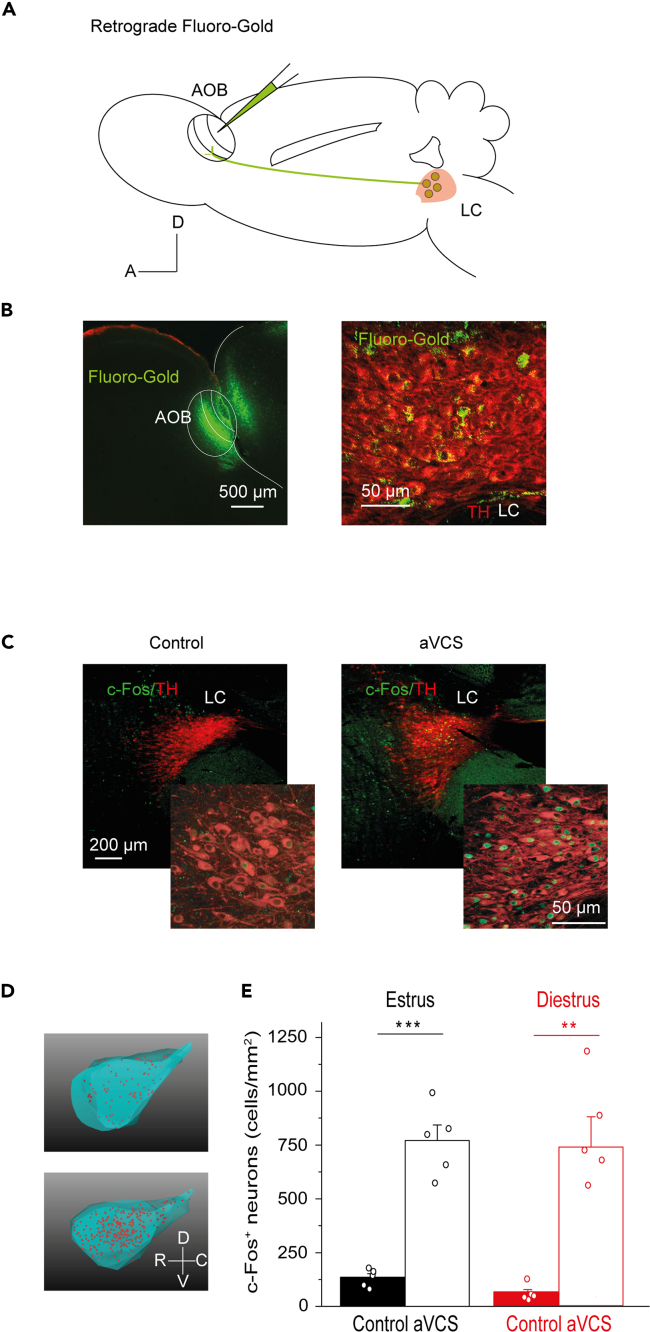
Locus coeruleus projects to the accessory olfactory bulb and is activated by artificial vagino-cervical stimulation in estrus and diestrus (A) Schematic of the experimental approach is shown. After iontophoretic infusion of tracer (Fluoro-Gold) into the AOB, retrogradely labeled cell bodies (green circles) were observed in the ipsilateral locus coeruleus (LC) (pink area). (B) Left panel shows infusion site (in green) in a sagittal AOB section. Right panel shows sagittal LC section with retrogradely labeled soma (green). Tyrosine hydroxylase (TH) positive noradrenergic neurons are in red. (C) Example of c-Fos immunostaining (green) in LC, in control (left) and 60 min after artificial vagino-cervical stimulation (aVCS; right). The insets show high resolution images. (D) Neurolucida reconstruction of c-Fos-positive neurons (red) in LC from estrous control (upper panel) and aVCS (lower panel) mice. (E) Quantification of c-Fos-positive neurons (mean ± SEM) in LC, in control and upon aVCS in estrus (black) and diestrus (red) are shown. c-Fos was significantly higher after aVCS in both estrus and diestrus (N = 5 per group; t-tests, ∗∗∗p < 0.001, ∗∗p < 0.01). Note that the level of c-Fos is higher in unstimulated estrous females compared to diestrus females, t-test, p < 0.05.

Projection from locus coeruleus to AOB in the mouse was confirmed by retrograde labeling with the fluorescent dye Fluoro-Gold (Figure 1A). The Fluoro-Gold iontophoretic infusion into AOB gave rise to labeled cells in the ipsilateral locus coeruleus (borders determined by tyrosine hydroxylase immunostaining; N = 4; Figure 1B). We then addressed if the first calibrated mechanical aVCS protocol, which is described herein (Figure S1), could recruit locus coeruleus. aVCS was delivered to anesthetized mice via a piezo-electrically controlled stimulator, placed in direct contact with the vaginal wall and cervix (Figures S1A-C). The vagino-cervical stimulator was shaped in 3D according to the anatomical features of the *Mus musculus* penis.^25^^,^^26^ The temporal stimulation pattern mimicked the natural thrust frequency during coitus.^27^^,^^28^ c-Fos was used as an activity marker, since electrical vagino-cervical stimulation has been reported to induce c-Fos in hypothalamus-projecting locus coeruleus neurons in the rat.^29^ The density of c-Fos positive cells in locus coeruleus (Figures 1C and 1D, cells/mm^2^) showed a significant > 3-fold increase after aVCS of estrous females compared to unstimulated controls (Figure 1E, Mann-Whitney U (MWU) test: median/quartile(Q)1/Q3: 800/616/910 after aVCS versus 145/90/171 in controls, p = 0.012. 770.6 ± 72.5 after aVCS versus 133.6 ± 18.7 in controls, N = 5, t-test: p = 2.8E-5). A similar increase was observed in diestrus (Figure 1E, MWU test: median/Q1/Q3: 680/465/1037 after aVCS versus 49/35/92 in controls, p = 0.012. Means: 737.1 ± 140.0 after aVCS versus 61.0 ± 17.3 in controls, N = 5, t-test: p = 0.001). Thus, aVCS increased c-Fos expression irrespective of the estrous phase (two-way ANOVA; p = 0.809). The baseline c-Fos was significantly higher (2-fold) in estrus compared to in diestrus (MWU-test, p = 0.037; t-test: p = 0.021).

Subsequently the effect of the aVCS protocol on local field potentials (LFPs) in the AOB mitral-tufted cell layer was characterized (Figure S1D). A downward LFP deflection was recorded upon aVCS. There was a graded stimulus-response relation between the amplitude of the stimulator and the evoked LFP response in AOB (Figures S1B, E). Signs of long-term response adaptation from the beginning to the end of the aVCS protocol, was not observed until the third stimulation block, suggesting that adaptation might not occur during the time frame of a typical mating sequence (Figure S1F). Thus, our calibrated aVCS protocol elicited a robust response in locus coeruleus from which AOB receives input, and AOB responded.

### Artificial vagino-cervical stimulation elicits a local field potential response largely mediated by α1-adrenergic receptors

Next, we tested if aVCS evoked an LFP response in the output layer of AOB and addressed if the α1-adrenergic receptor was involved. We took advantage of the layered organization of AOB, where the somata of the mitral-tufted output neurons are separated from the somata in the glomerular and GC layers (Figure 2A), and LFP recordings can discriminate responses generated in these layers.^30^ First, LFP responses to aVCS were recorded in the mitral-tufted cell layer. The nominal depth of the electrode was within a spatial range of the mitral-tufted cell layer (median/Q1/Q3: 1300/1250/1350 μm; N = 30) and localization was confirmed by histological examination of the DiI-labeled electrode track (Figure 2A). LFP recorded in the mitral-tufted cell layer conceivably reflected the combined activities in the mitral-tufted neuron dendrites and the reciprocal synapses between these neurons and GCs.^21^^,^^30^^,^^31^

aVCS gave rise to stimulus-locked LFP responses, which were invariably downward in estrus (Figure 2C) as well as in diestrus (Figure 2D). These aVCS-evoked potentials (aVCS-EPs) were over in about 2 s and apparently shorter than the duration of each 5 s stimulatory block. *In vitro* responses to norepinephrine can be significantly longer.^16^^,^^18^ Note that LFPs report mainly transient changes in extracellular net current, so a tonic change in the homeostatic equilibrium between excitation and inhibition of the reciprocal synapses is not expected to be detected. There was no difference in aVCS-EPs between estrus and diestrus (N = 7 and 5, respectively) with regard to the amplitude (77 ± 11 versus 82 ± 14 μV; MWU-test, p = 0.620; t-test, p = 0.790) or latency (616 ± 99 versus 757 ± 267 msec; MWU-test, p = 1.000; t-test, p = 0.580). Notably, aVCS-EPs were not detected before the electrode entered the AOB, i.e., there was no response in prefrontal cortex (Figure 2B) and neither in the main olfactory bulb (N = 5 mice for each group; Figure 2E). The latter result may be related to mounting evidence pointing to a high degree of functional heterogeneity in terms of input/output connectivity of different partitions of locus coeruleus.^32^

To address if the aVCS-EPs were dependent on norepinephrine that can be released from locus coeruleus, aVCS-EPs were measured before and after local infusion of the selective α1-adrenergic receptor blocker WB4101 in estrus (0.5 mL 30 μM; Figures 2F and 2G). WB4101 significantly reduced the peak amplitude of aVCS-EPs compared to vehicle (median/Q1/Q3 vehicle control: pre-infusion 82.5/66.3/97.9 versus post-infusion 94.3/35.3/116.3 μV, Wilcoxon signed-rank (WSR) test, p = 0.385. WB4101: pre-infusion 137.1/72.9/156.5 versus post-infusion 66.1/44.9/100.3 μV, WSR-test, p = 0.029; N = 5 for each). Parametric tests gave similar results (Vehicle control: pre-infusion amplitude 83.0 ± 8.6 versus 83.7 ± 17.4 μV post-infusion. WB4101: pre-infusion 119.2 ± 19.4 versus 71.3 ± 13.9 μV post-infusion; paired t-tests p = 0.590 and p = 0.020, respectively).

Taken together, aVCS gave rise to a graded, stimulus-locked LFP response in the AOB mitral-tufted cell layer in estrus as well as in diestrus. Moreover, the aVCS-EP response was to a significant extent dependent on the activation of α1-adrenergic receptors as shown in estrous females.

### Estrus-specific prolonged activation of the inhibitory granule cell layer

**Figure 3:**
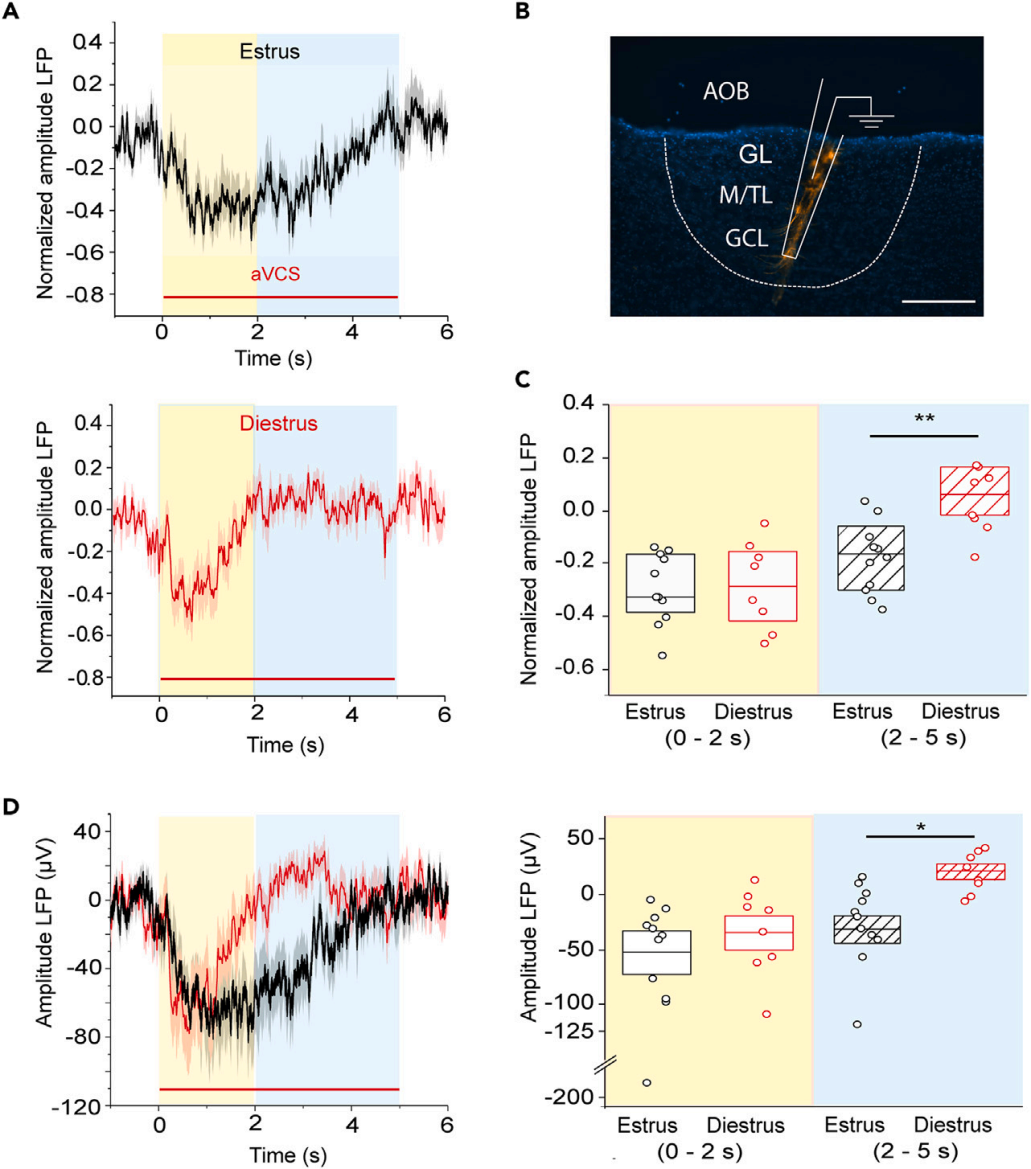
Artificial vagino-cervical stimulation results in prolonged activation of the inhibitory granule cell layer in estrus females (A) The normalized grand average of aVCS-EPs recorded in the GC layer in estrus (black line, upper panel, N = 11 mice) and diestrus (red line, in lower panel, N = 8 mice), is shown. The thick lines are grand averages, while the thin shadows represent SEMs. Note that 2 sec from onset of the 5 sec aVCS, the signal is back to baseline in diestrus, while it lasts longer estrus. (B) Sagittal AOB section showing a trace of DiI-labeled electrode (orange) targeted to the GC layer (blue nuclear staining, DAPI). Scale bar: 200 μm. (C) Normalized median amplitudes in two aVCS time windows. In the early phase (0–2 sec; yellow area) there was no difference in the response in estrus compared to diestrus. Note that in estrus the activation of the GC layer lasted into the later time window (2–5 ec, light blue area) while this was not the case in diestrus (MWU-test, ∗∗p < 0.01). Boxplots represent 25th percentile, median and 75^th^ percentile from bottom to top. (D) Left shows same type of plot as in (A) but with overlay of absolute aVCS-EPs in estrus (black) and diestrus (red). Right shows absolute voltage values and can be compared to (C), which shows normalized data (MWU-test, ∗p < 0.05).

Next LFP recordings were made from the GC layer, as previous studies in slices suggested that norepinephrine from locus coeruleus activates GCs.^33^ LFP recordings were obtained on average at a 300 μm deeper nominal depth compared to recordings from the mitral-tufted cell layer (median/Q1/Q3: 1600/1500/1725 μm; N = 19; Figure 3B). In diestrus the aVCS-EP was over after 2 sec of stimulation, which was similar to the time frame for the response in the mitral-tufted layer (Figure 3A). Previous slice studies *in vitro* indicated that GCs analyzed from pre- and peripubertal males and females, do not appear to have a cell intrinsic adaptation mechanism to norepinephrine during a > 5 sec stimulus period^16^^,^^18^ and thus the GC adaptation during the 5 sec single aVCS sweep might be an *in vivo* emergent property.

Most intriguingly, in estrous females the GC layer response clearly outlasted the response in diestrus, as the peak was at around 2 sec instead (time windows in yellow and blue, respectively Figure 3A). To quantify this difference, median amplitudes of normalized aVCS-EPs in the two aVCS time windows were compared (early: 0–2 sec; late: 2–5 sec; Figure 3C). aVCS-EP amplitudes were statistically comparable during the early activation in both conditions (estrus median/Q1/Q3: −0.33/-0.40/-0.16, N = 11; diestrus: −0.27/-0.44/-0.14, N = 8. MWU-test, p = 0.710). Conversely, aVCS-driven activation of the GC layer showed larger amplitudes in estrus compared to diestrus in the later time window (estrus median/Q1/Q3: −0.15/-0.28/-0.03; diestrus: 0.08/0.00/0.18. MWU-test, p = 0.004). Analysis of the same, but not normalized data, confirmed this result (Figure 3D).

Thus, the first AOB microcircuit location where we could demonstrate an estrous cycle-dependent difference in the response to aVCS, was the GC layer, which showed a difference in temporal activation dynamics.

### Mitral-tufted neurons are relatively hyperpolarized and spontaneously fire high frequency bursts in estrus

**Figure 4:**
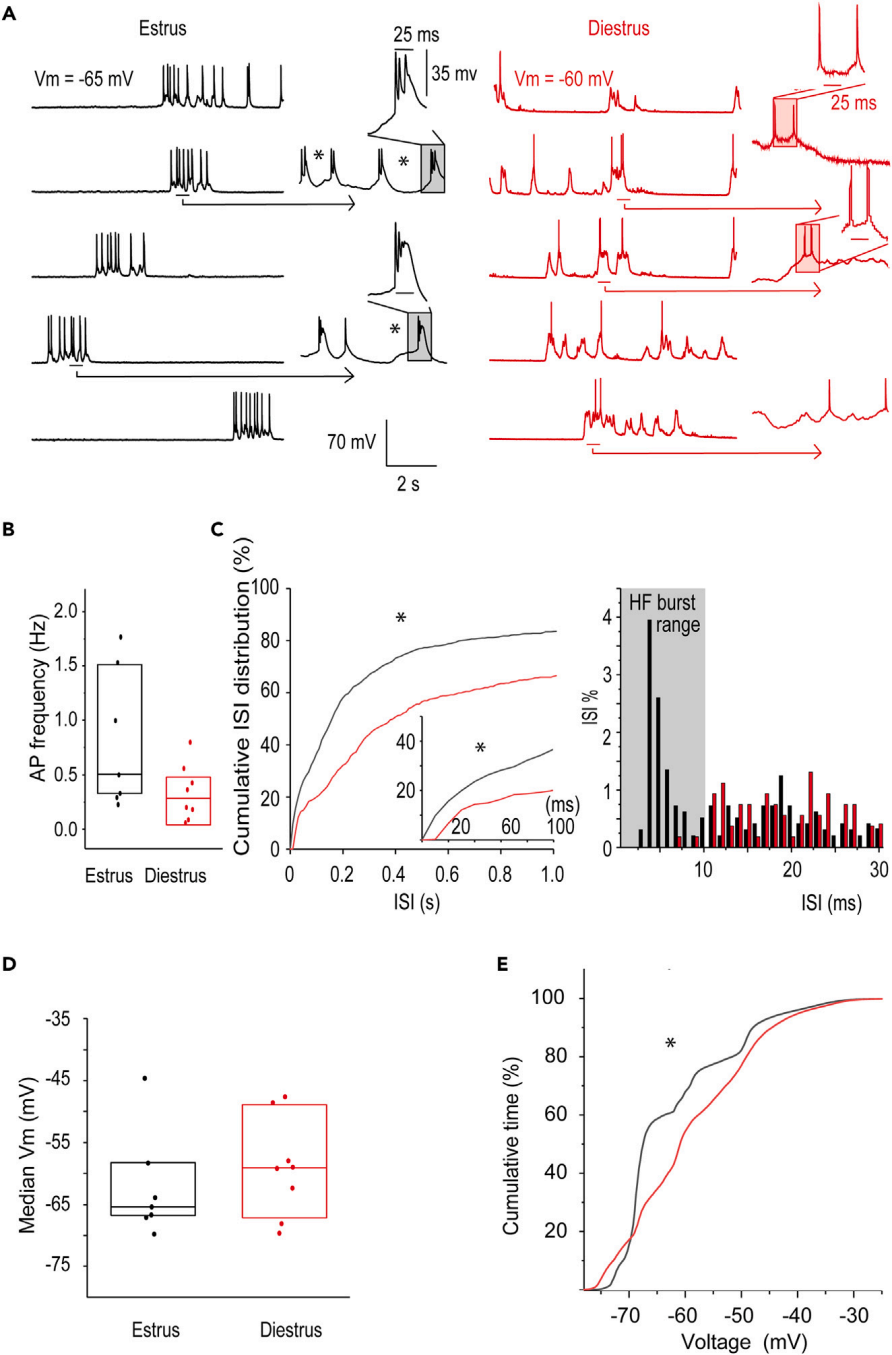
During estrus mitral-tufted neurons spontaneously fire actions potentials in high frequency bursts from a more hyperpolarized resting V_m_ (A) Examples of spontaneous activity (i.e., without aVCS) from single mitral-tufted neurons in estrus (left) and diestrus (right) captured by intracellular patch clamp recording, are shown. To visualize spikes that are very close in time, windows of 500 msec (horizontal bars), have been magnified. Spikes close in time have been highlighted and magnified further. ∗ Denotes examples of two spikes that are ≤ 10 msec apart. Note that in these traces only recordings in estrus demonstrate spikes with ≤ 10 msec intervals, which are of an HF burst type. (B) Action potential (AP) frequency of mitral-tufted neurons is shown. Despite the tendency for higher firing rates in estrus, the difference did not reach statistical significance (MWU-test, p = 0.105). (C) Close up of quantitative analysis of the cumulative distributions of interspike intervals (ISIs) measured in estrus (black line) and diestrus (red line) is required to show differences in HF bursting in estrus versus diestrus. The distribution for estrus was significantly skewed toward shorter ISIs (∗ K-S-test, p < 0.001). The same was true when analysis was restricted to action potential frequencies of 1kHz to 10Hz, corresponding to ISIs 1 to 100 msec, respectively, (inset). Right histogram shows percent ISI at high resolution in the bursting range 1–30 msec. Note that ISIs in the HF bursting range of ≤ 10 msec (gray shading) are almost exclusive to estrus. (D) Median V_m_ values for estrus and diestrus were comparable. (E) The subthreshold cumulative distribution showed that individual neurons spent more time in a hyperpolarized state in estrus, compared to in diestrus (∗K-S-test, p < 0.001). Data were from: n = 7 neurons from N = 7 estrous mice; n = 8 neurons from N = 7 diestrous mice. In B and D boxplots represent 25th percentile, median and 75^th^ percentile from bottom to top.

To explore if the estrous cycle phase *per se* influenced the ongoing activity or the intrinsic cell excitability, *in vivo* whole-cell recordings from mitral-tufted neurons were performed in absence of stimulation (Figure 4A; additional examples in Figure S2).

Suprathreshold activity (i.e., spike output) was analyzed first. There was a trend toward an increase of spontaneous action potential frequency in estrus (median/Q1/Q3: 0.54/0.30/1.53 Hz) compared to diestrus (0.28/0.10/0.52 Hz), but significance was not reached (Figure 4B MWU-test, p = 0.105; n = 7 estrus and n = 8 diestrus from N = 7 and N = 8 mice, respectively).

Mitral-tufted neurons displayed different temporal patterns of spiking depending on estrous cycle phase, which were quantitatively analyzed as the frequencies of different interspike intervals (ISIs, in msec^34^; Figure 4C graph+inset and histogram). Despite the statistically comparable average action potential firing frequencies, the median ISIs were shorter in estrus (median/Q1/Q3 = 1.13/0.56/3.14 sec) compared to in diestrus (3.58/1.90/8.65 sec; MWU-test p = 0.032). Thus, the cumulative distribution of all recorded ISIs in estrus was skewed toward lower values relative to the ISI result for diestrus (Figure 4C Kolmogorov-Smirnov (K-S) test; p = 2.11E-21). Further analysis of ISIs focused on the interesting 1–100 msec range (corresponding to 1000-10 Hz; inset Figure 4C, left), indicated that there were estrous cycle-dependent differences within the ISI range of 0–30 msec, which is well within the range where neurons are considered to display burst firing. As the temporal resolution was increased (Figure 4C, histogram on the right) it became evident that ISIs of less than 10 msec were virtually exclusive to neurons during estrus. For the sake of clarity ≤ 10 msec ISIs are hereafter termed to signify firing within a high frequency bursting range (HF bursting;^35^). The percent ≤ 10 msec ISIs per neuron was significantly higher for neurons during estrus (median estrus: 2.3% and diestrus 0.0%, MWU-test, p = 0.011).

We next analyzed the subthreshold activity. Membrane potential (V_m_) values were not statistically different for estrus when calculating medians for each neuron (median/Q1/Q3: −65.4/-66.7/-58.3 mV) compared to values for diestrus (−59.1/-66.6/-50.8 mV; MWU-test, p = 0.452; Figure 4D). A more sensitive analysis to capture differences in resting V_m_ values, is by analyzing the cumulative distributions of the time spent at different V_m_ values, which were quantified and distributions compared. This analysis showed that neurons spent significantly more time in a hyperpolarized resting state in estrus than in diestrus (Figure 4E K-S-test, p = 7.75E-4). The action potential threshold is another important determinant of cell intrinsic excitability. Values were computed as the point of maximal acceleration of V_m_ right before each spike top and this revealed that neurons in estrous females had more hyperpolarized action potential threshold values (median/Q1/Q3: −40.5/-45.1/-38.3 mV) compared to females in diestrus (median/Q1/Q3: −32.0 mV/-36.6/-29.6 mV; MWU-test, p = 0.018).

Together these results indicated that in estrus, neurons in the mitral-tufted layer were relatively hyperpolarized and had a greater propensity to fire action potentials in HF bursts.

### A vagino-cervical stimulus-evoked inhibitory synaptic mechanism reduces mitral-tufted neuronal output specifically in estrus

**Figure 5:**
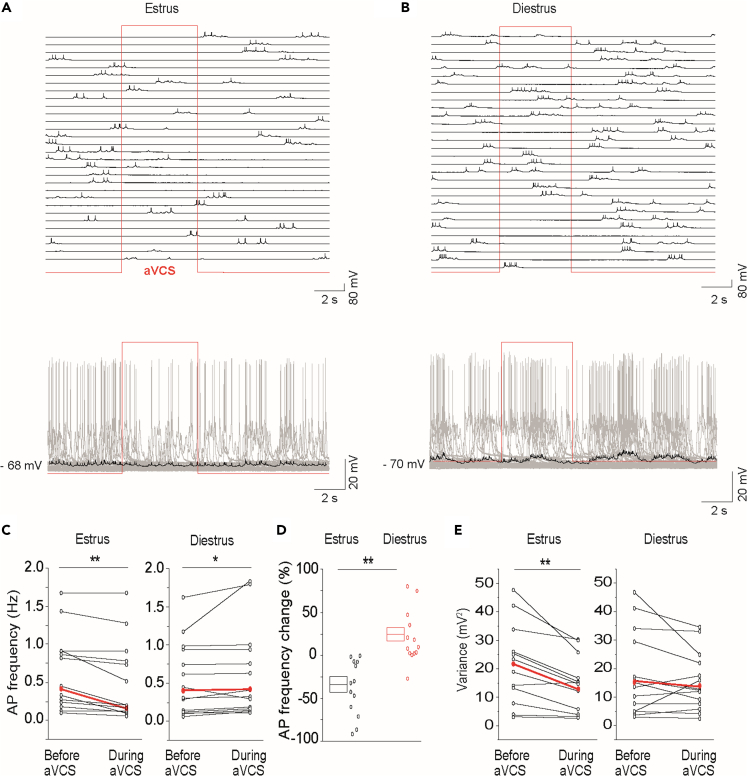
Estrus-specific artificial vagino-cervical stimulus-evoked reduction of spike output and membrane resistance of mitral-tufted neurons (A) Example of *in vivo* whole-cell recording of neurons from the mitral-tufted layer showing inhibitory modulation of ongoing activity by aVCS in estrus (top panel, traces of single sweeps; bottom panel, overlaid traces). (B) Shows example of recording in diestrus (top panel, traces of single sweeps; bottom panel, overlaid traces). (C) Quantitative analysis of spiking is required to analyze the effect of aVCS. Paired quantification of the mean action potential (AP) firing output frequency before and during aVCS, in estrus and in diestrus, is shown. aVCS induced a significant decrease in AP in estrus, but not in diestrus, which instead showed a slight significant increase (estrus: N = 12, n = 13, WSR-test, ∗∗p < 0.01; diestrus: N = 12, n = 14, WSR-test, ∗p < 0.05). (D) Shows percent modulation of the firing rate calculated for each recorded neuron. A significant difference was found between estrus and diestrus (t-test, ∗∗p < 0.01). Boxplots represent means±SEMs. (E) Analysis of the V_m_ variance (mV^2^) change before and during aVCS in estrus (left) and diestrus (right) is shown. Result indicates that aVCS may activate synaptic inhibition in mitral-tufted neurons largely restricted to estrus. The plots show the mean values of all the sweeps (one pair of values per recorded neuron) before and after aVCS (paired t-tests, ∗∗p < 0.01 for estrus).

Next the synaptic (subthreshold) and spike (suprathreshold) nature of the aVCS response was investigated by *in vivo* whole-cell recordings of mitral-tufted neurons. An inhibitory response might be expected in estrus given that the GCs showed prolonged activation by aVCS (Figure 3D). In estrus a decrease of spike output during aVCS was observed in the majority of recorded neurons (10 out of 13; Figures 5A and 5C; Figure S3 example of not inhibited neuron). The quantification of the spike rate modulation in response to aVCS in estrus and diestrus is given by the paired comparison before and during aVCS (Figure 5C). This analysis revealed a significant stimulus-driven negative modulation of the spiking frequency in estrus (Figure 5C, left). At population level, median firing rate before aVCS was 0.36 Hz (Q1/Q3: 0.12/0.84), whereas the rate during aVCS was 0.16 Hz (Q1/Q3: 0.06/0.79 Hz; N = 12 mice, n = 13 cells; WSR-test, p = 0.002). Importantly, recordings in diestrus did not show a decrease in the mean action potential firing rate at population level during aVCS (Figure 5B, quantification in Figure 5C right). Median firing frequency before aVCS was 0.37 Hz (Q1/Q3: 0.12/0.95 Hz) and during aVCS 0.38 Hz (Q1/Q3: 0.17/0.96 Hz; n = 14 cell, N = 12 mice; WSR-test, p = 0.01). Two-way mixed-design ANOVA showed a significant interaction of aVCS and estrus state (p = 0.006). In similarity to experiments shown previously for the spontaneous firing frequencies, there was no difference in the prestimulus values in estrus versus diestrus (MWU-test, p = 0.942). Thus, this indicates that the absence of aVCS-induced spike inhibition in diestrus, is not due to a generally lower activity level in diestrus. In line with the inhibitory modulation of spike output by aVCS specifically in estrus, the paired analysis of the mean firing rates before and during stimulation revealed that aVCS caused a 36.7 ± 10.2% mean decrease in estrus, whereas in diestrus aVCS elicited a mean increase of 23.3 ± 9.6%. The relative changes in spike frequency between estrus and diestrus were significantly different (Figure 5D MWU-test median/Q1/Q3: −36.3/-64.2/-2.3 versus 10.4/2.1/45.8 for estrus and diestrus, respectively, p = 4.2E-5; t-test, p = 2.4E-4).

Reduction in spike rate could be due to increased inhibition or withdrawal of excitation. Thus, next we addressed the synaptic basis for the estrus-specific relative spike output reduction in response to aVCS. There was no significant change in the resting V_m_ during aVCS in estrus (average V_m_ before aVCS median/Q1/Q3: −66.8/-57.9/-70.9 mV; V_m_ during aVCS: −67.1/-56.9/-71.0 mV; WSR-test, p = 0.64, n = 13). Likewise, V_m_ did not change in diestrus (V_m_ before aVCS median/Q1/Q3: −62.8/-53.7/-68.7 mV; V_m_ during aVCS: −62.8/-50.4/-68.4 mV; WSR-test, p = 0.067, n = 14). It is not trivial to measure sensory stimulation-driven pure hyperpolarizations *in vivo* as e.g., the resting V_m_ is close to the equilibrium potential for Cl^−^ and K^+^, and there is simultaneous activation of excitatory and inhibitory synapses. It is particularly difficult to measure inhibition when, like in our case, the origin of the inhibition is distant from where recordings are done, at the soma level.^36^

Inhibition by opening GABA-channels should cause a decrease in membrane resistance so that the same synaptic currents impinging on a neuron result in smaller V_m_ deflections (i.e., reduced V_m_ variance over time). Withdrawal of excitation should result in the opposite. Analysis of variance for the aVCS-induced spike reduction was done by searching the mean of variance in a temporal window (±500 msec) around the local minimum of firing during aVCS. aVCS induced a statistically significant decrease of V_m_ variance specifically in estrus (Figure 5E left; n = 13, WSR-test median/Q1/Q3: 18.8/10.4/29.9 versus 12.6/5.3/21.0 mV^2^, before and during aVCS, respectively, p = 0.006. 21.0 ± 3.8 versus 14.2 ± 2.6 mV^2^, paired t-test, p = 0.002), but not in diestrus (Figure 5E right; n = 14; WSR-test median/Q1/Q3: 12.5/2.8/29.6 versus 11.3/3.7/21.1 mV^2^, before and during aVCS, respectively, p = 0.258. 15.9 ± 4.1 mV^2^ versus 13.6 ± 2.8 mV^2^, paired t-test, p = 0.318). Furthermore, computation of conductance modulation contrast showed significant difference between estrus and diestrus (contrast defined as difference of variances before and during aVCS divided by their sum; medians: 0.21 versus 0.09, MWU-test, p = 0.055; means: 0.17 ± 0.02 versus −0.01 ± 0.09, t-test, p = 0.035). Paired analysis of the same dataset, but on a single sweep-basis, also showed a significant decrease in variance only in estrus (estrus before stimulation: median/Q1/Q3: 2.7/0.2/23.9 mV^2^ versus during aVCS 0.8/0.2/12.8 mV^2^, WSR-test, p = 7.2E-4. diestrus before stimulation: 0.6/0.1/15.0 mV^2^ versus during aVCS 0.7/0.1/14.0 mV^2^, WSR-test, p = 0.478). These results demonstrated that the estrus-specific prolonged GC activation by aVCS was accompanied by a reduction in the spontaneous ongoing spike output of mitral-tufted neurons caused by an inhibitory synaptic mechanism akin to that triggered by local GABA release.

### Interfering with dendro-dendritic communication via reciprocal synapses prevents artificial vagino-cervical stimulus-driven spike inhibition and estrus-specific bursting

**Figure 6:**
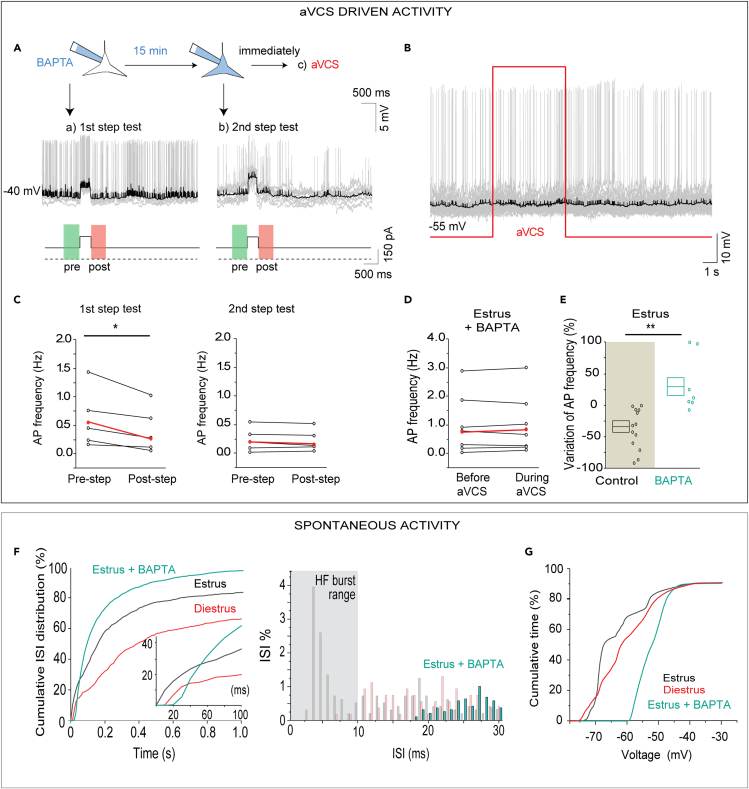
Artificial vagino-cervical stimulus-driven inhibition relies on dendro-dendritic communication (A) The experimental protocol is outlined. A background current of +150 pA was constantly injected to induce the neuron to fire during each test pulse. Top: right after whole-cell stabilization, a test pulse was given to the mitral-tufted neuron to be able to measure possible spike reduction right after this pulse step, as a measure of inhibition via the reciprocal synapses between mitral-tufted and GC dendrites. After 15 min the test was repeated ± BAPTA and was then immediately followed by aVCS. Traces represent examples of the test pulses (10 sweeps overlaid). (B) Example of activity during aVCS in a BAPTA-perfused neuron. (C) Paired comparisons of the mean firing rate observed in the pre- and post-step time windows (highlighted in green and red in A). A significant decrease was observed right after 1^st^ step test, before dendritic diffusion of BAPTA, but not after 2^nd^ step test when BAPTA had been allowed diffusion for 15 min within the very same neurons (N = 5 mice, and n = 5 cells, paired t-tests, ∗p < 0.05). (D) Paired plots show the absence of aVCS-driven spike decrease by aVCS when BAPTA was present (n = 7 neurons). (E) Shows lack of percentual decrease of mean firing rates during aVCS compared to prestimulus time in estrus animals under BAPTA, compared to estrus controls (MWU-test, ∗∗p < 0.01). Note that the estrus control from Figure 5D is replotted for comparison (shaded). Boxplots represent means±SEMs. (F and G) Spontaneous activity recorded during the influence of BAPTA is shown. (F) Cumulative distributions of the ISI range for the three experimental groups of neurons recorded from estrus, diestrus or estrus+BAPTA are compared. The estrus+BAPTA group shows a curve which is statistically different from both estrus and diestrus groups (K-S-test, p < 0.001). Inset shows curves for the interesting 1–100 msec ISIs range. Histogram to the right shows percent ISI with 1 msec resolution in the < 30 msec bursting range. Note that intracellular BAPTA abolishes estrus enriched HF bursts (≤ 10 msec, gray shading). (G) The cumulative distributions of time spent at different membrane potentials show that in presence of intracellular BAPTA, neurons were significantly more depolarized compared neurons in estrous as well as diestrous mice (K-S-test, p < 0.001).

To address whether reciprocal synapses between mitral-tufted neurons and GCs might be required for the aVCS-driven spike reduction in mitral-tufted neurons observed in estrus, we used an intracellular calcium chelator (BAPTA).^36^^,^^37^^,^^38^^,^^39^^,^^40^ Whole-cell recording of action potentials was started right after patch rupture in such a way that spike rate measurements were done before and after a depolarizing step that drove the mitral-tufted neuron to spike (Figure 6A, 1^st^ step test; pre-step firing rates: median/Q1/Q3: 0.47/0.31/1.02 versus post-step: 0.16/0.03/0.70, WSR-test p = 0.029; N = 5 mice, n = 5 cells. Mean firing frequency pre-step was 0.55 ± 0.23 Hz and post-step 0.32 ± 0.17 Hz; paired t-test, p = 0.021; Figure 6C, left). Thus, electrically induced spiking of mitral-tufted neurons apparently stimulated the GCs to inhibit the mitral-tufted neurons via the reciprocal synapse. This step test was repeated after 15 min, which allowed for diffusion of BAPTA throughout dendrites (Figure 6A, 2^nd^ step test). At this time point, the median firing frequencies were statistically comparable (WSR-test pre-step median/Q1/Q3: 0.18/0.03/0.42 versus post-step 0.09/0.04/0.27, p = 0.281. Mean firing frequencies pre-step was 0.21 ± 0.09 Hz and post-step 0.14 ± 0.06 Hz paired-sample t-test, p = 0.122. Figure 6C, right).

The involvement of the reciprocal synapse in aVCS-induced inhibition of mitral-tufted neuron spikes could then be addressed. Figure 6B exemplifies that aVCS-driven spike reduction was no longer apparent 15 min after the start of BAPTA infusion, as there was no difference in median firing rates, which were 0.69 Hz (Q1/Q3 0.08/1.90 Hz) before aVCS and 0.70 Hz during aVCS (Q1/Q3 0.29/1.7 Hz; N = 7 mice, n = 7 cells; WSR-test, p = 0.125. Figure 6D). As shown previously (Figure 5D), aVCS caused a decrease in firing rate (medians/Q1/Q3: −36.3/6.27/-2.3%), but when the neurons were infused with BAPTA this did not occur (11.5/0.71/-99.0%, MWU-test p = 0.002. Means: −36.7 ± 10.2% versus +32.6 ± 17.9%; t-test, p = 0.002. Figure 6E).

We also analyzed if the spontaneous activity in estrus was influenced by BAPTA. The ISI cumulative distribution for the estrus+BAPTA group was different from both estrus and diestrus (K-S-test: estrus versus estrus+BAPTA, p = 1.42E-17; diestrus versus estrus+BAPTA, p = 3.69E-64. Figure 6F). Of relevance, the estrous-specific HF bursts (ISIs ≤ 10 msec) were absent in neurons subjected to intracellular BAPTA (Figure 6F, right histograms). The percent ≤ 10 msec ISIs per neuron after intracellular BAPTA was negligible in estrus and statistically indistinguishable from diestrus (median diestrus: 0.0% and estrus+BAPTA 0.0%, MWU-test, p = 0.772). 10–20 msec ISIs were also absent after BAPTA. The cumulative distribution of the time neurons spent at various V_m_ values showed that neurons in the estrus+BAPTA group were relatively depolarized compared to neurons recorded in estrus or in diestrus (K-S-test: estrus versus estrus+BAPTA, p = 6.29E-15; diestrus versus estrus+BAPTA, p = 1.15E-13. Figure 6G).

The depolarizing effect of BAPTA observed under control conditions would be expected to increase the driving force for inhibitory current by either Cl^−^ or K^+^ also during aVCS. These biophysical conditions would favor inhibition, but aVCS-driven spike reduction was instead prevented by BAPTA, which is in line with spike reduction being dependent on the reciprocal synapses.

Altogether, these data indicated that both the aVCS-driven spike reduction and the spontaneous HF bursting observed in estrus required the integrity of the reciprocal synapse.

### Artificial vagino-cervical stimulation-driven spike inhibition and bursting are not observed under α1-adrenergic receptor blockade during estrus

**Figure 7:**
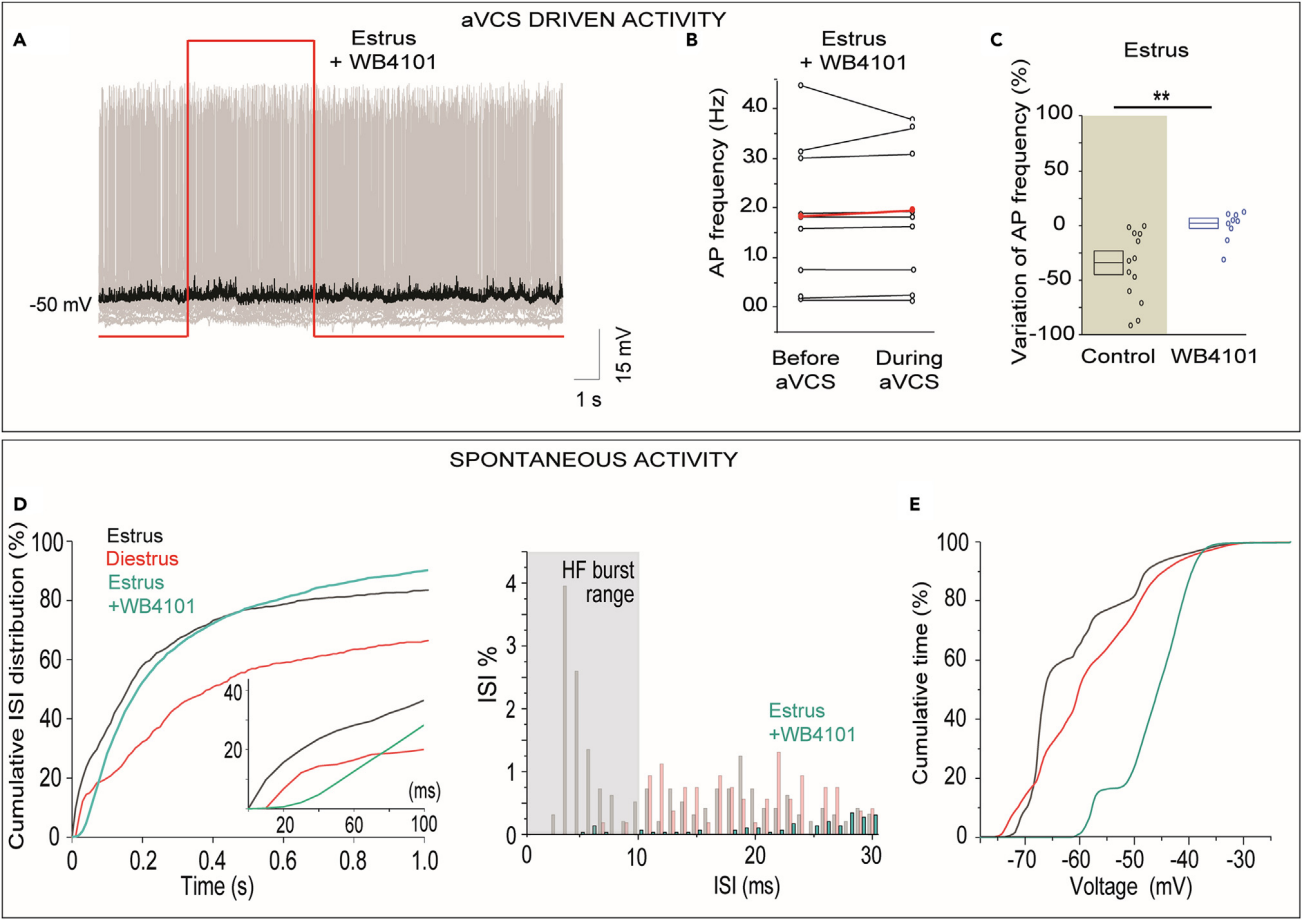
α1-adrenergic receptor blockade prevents the artificial vagino-cervical stimulus-driven inhibition of mitral-tufted neuronal activity (A) Example (overlaid sweeps) shows that aVCS-driven spike reduction in estrus did not occur after extracellular α1-adrenergic receptor blocker WB4101. (B) Paired plot shows lack of significant decrease of mean firing rates during aVCS in the presence of WB4101 (N = 5 mice in estrus, n = 9 cells). (C) Shows percentage change of the mean firing rate. The receptor blocker prevented the otherwise observed spiking inhibition during aVCS (MWU-test, ∗∗p < 0.01). Note that the estrus control from Figure 5D is replotted for comparison (shaded). Boxplots represent means±SEMs. (D and E) Spontaneous activity during α1-adrenergic receptor blockade is shown. (D) Cumulative distribution of the ISI for three experimental groups. The estrus+WB4101 curve is statistically different from both estrus and diestrus curves (K-S-tests, p < 0.001). Inset shows cumulative plots in the 1–100 msec range. Histogram to the right shows percent of ISI with 1 msec resolution in the 1–30 msec range. Note that extracellular α1-adrenergic receptor blockade abolishes HF bursts (ISIs ≤ 10 msec, gray shading). (E) The cumulative distributions of time spent at different membrane potentials show that neurons in estrous mice subjected to blocker WB4101 were significantly more depolarized than neurons in estrous and diestrous mice (K-S-test, p < 0.001).

The results aforementioned suggested that the estrus-specific aVCS response in GCs was required for eliciting aVCS-driven spike inhibition in mitral-tufted neurons. If so the inhibition should be sensitive to extracellular pharmacological blockade of α1-adrenergic receptors. Our population data for firing rates revealed that aVCS did not cause a spike inhibition in mitral-tufted neurons when the blocker WB4101 was present, while spike inhibition was evident in controls (Figures 7A–7C). Median firing rates in estrous animals with WB4101, was 1.75 Hz (Q1/Q3 0.41/3.05 Hz) before stimulation and 1.85 Hz during aVCS (Q1/Q3 0.43/3.30 Hz; N = 5 mice, n = 9 cells; WST-test, p = 0.286. Figure 7B). WB4101 blockade of the aVCS-driven decrease of firing rates was also evident when comparing the percent change in firing in response to stimulation (MWU-test: median/Q1/Q3: −36.3/-64.2/-2.3 versus 5.4/-6.6/7.7 for without versus WB4101, respectively; p = 0.002; means: −36.7 ± 10.2% in control versus 0.8 ± 4.7% with WB4101; two-sample t-test, p = 0.009. Figure 7C).

In addition, we tested whether α1-adrenergic receptor blockade affected the estrous-specific spontaneous spike pattern. The cumulative distribution of ISI values for the estrus+WB4101 group was different from both estrus and diestrus groups (Figure 7D K-S-tests: estrus versus estrus+WB4101, p = 2.69E-24 and diestrus versus estrus+WB4101, p = 5.11E-24). Importantly, HF bursts (ISI ≤ 10 msec) were almost completely abolished by WB4101 (Figure 7D, histogram to the right). 10–20 msec ISIs were also absent under WB4101 blockade. The percent ≤ 10 msec ISIs per neuron in presence of WB4101 was negligible in estrus and statistically indistinguishable from diestrus (median diestrus: 0.0% and estrus+WB4101 0.0%, MWU-test, p = 0.638). Thus, this result implied α1-adrenergic receptor activation in the regulation of the propensity of mitral-tufted neurons to fire in HF bursts spontaneously during estrus.

At subthreshold level, neurons recorded under α1-adrenergic receptor blockade were more depolarized than neurons during estrus or diestrus (estrus+WB4101 resting V_m_ median/Q1/Q3: −45.7/48.6/-41.3 mV; estrus resting V_m_ median/Q1/Q3: −65.4/-66.7/-58.3 mV; diestrus resting V_m_ median/Q1/Q3: −59.1/-66.6 mV/-50.8 mV; Kruskal-Wallis ANOVA-test of estrus+WB4101/estrus/diestrus, p = 0.005). The cumulative distributions of time spent at various V_m_ for these three experimental groups confirmed that result (K-S-tests: 1) estrus versus estrus+WB4101, p = 6.3E-15, 2) estrus versus diestrus, p = 7.75E-4, 3) diestrus versus estrus+WB4101, p = 3.24E-10. Figure 7E).

Note, as discussed previously for BAPTA, the more depolarized V_m_ values observed also under the α1-adrenergic receptor blockade theoretically should increase the driving force for inhibitory currents and thus facilitate the experimental detection of aVCS-driven spike inhibition, which WB4101 instead prevented (Figure 7A).

## Discussion

By using a technical approach with cell-type resolution that allows for distinguishing sub- and suprathreshold events *in vivo*, we find specific differences in the ongoing activity of the AOB mitral-tufted output neurons in estrus versus diestrus. In estrus, mitral-tufted neurons were relatively hyperpolarized and fired action potentials in HF bursts. Moreover, this is the first study of single mitral-tufted neuronal responses to calibrated vagino-cervical stimulation at the microcircuit level, in naturally cycling females.

**Figure 8:**
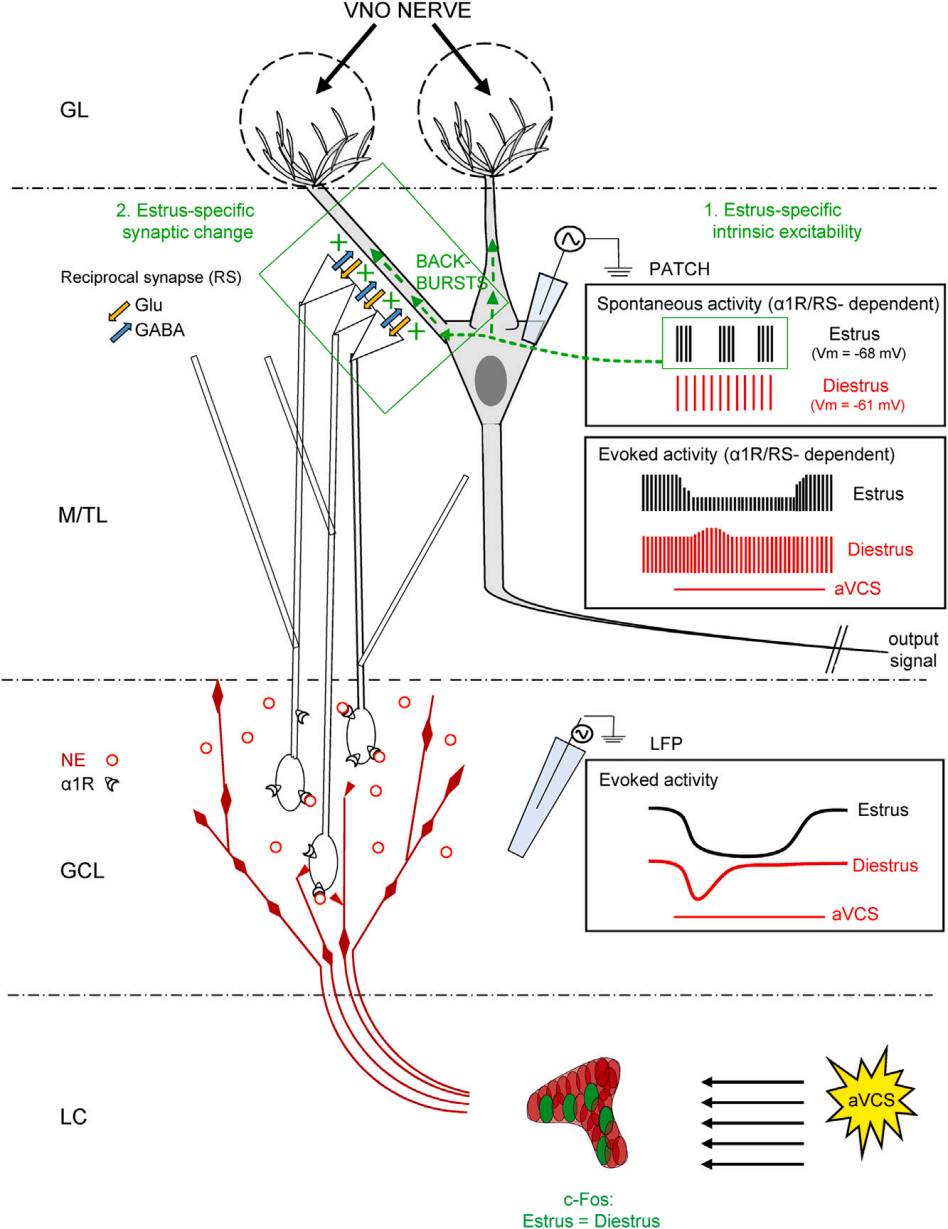
Model of AOB microcircuit basis for estrus-specific high frequency bursting and artificial vagino-cervical stimulus-driven spike inhibition, in output neurons Starting at the bottom of the illustration, the locus coeruleus (LC) responds to aVCS by increased activity as assessed by marker c-Fos. Similar numbers of LC neurons get activated in estrous and in diestrous mice. LC fibers should then increase release of norepinephrine (NE) in the granule cell soma layer (GCL), which activates α1-adrenergic receptors (α1R). aVCS-evoked activity, measured by LFP in the GCL, shows a prolonged activation of GCs in estrus compared to in diestrus. This estrus-specific prolonged aVCS response in GCs should result in extended release of GABA at the reciprocal synapse (RS) between mitral-tufted (M/T) neurons and GCs, which are located in the M/T layer (M/TL; see green box). The longer activation of GCs conceivably leads to the reduced spiking we find in M/T glutamatergic output neurons during estrus. Thus, the output activity from the M/T neurons in AOB, in response to a coital stimulus, depends on the estrous state of the female. Intracellular blockade of the RS by BAPTA disrupts the estrous-specific spike inhibition, and this suggests that the RS is central to the estrus-specific microcircuit behavior. Moreover, we find that the spontaneous activity of M/T neurons, in the absence of stimulation, also depends on the estrous cycle phase. M/T neurons are relatively hyperpolarized in estrus compared to diestrus (medians from Figure 4E). There also is an ongoing high frequency (HF) spike bursting of M/T neurons during estrus (indicated by 1). These HF bursts likely back-propagate (BACK-BURSTS) in M/T dendrites and should more effectively stimulate the RSs (indicated by 2 and +). We propose that the coincidence of increased spontaneous stimulation of the M/T membrane part of the RS, and the arrival of aVCS-evoked, NE-induced depolarization of the GC membrane part of RS, is a candidate mechanism to underlie the estrus-specific aVCS-induced spike inhibition in M/T neurons that is shown herein. See discussion for the potential contributions of NMDA and mGluR receptors to the prolonged activity of GCs in estrus.

Extracellular recordings reveal that aVCS induces a stimulus-locked and graded LFP response in the GC layer that is largely mediated by the α1-adrenergic receptor. The duration of this aVCS-EP is longer in estrus than in diestrus. This prolonged GC response to aVCS is accompanied by estrous-specific spike inhibition in the mitral-tufted neurons as recorded intracellularly. The reciprocal synapses between GCs and mitral-tufted neurons are required for the aVCS-evoked relative inhibition of mitral-tufted neuronal spike output, which is observed in estrus. The reciprocal synapses appear central also to the observed differences in spontaneous mitral-tufted neuronal activities in estrus versus diestrus (Figure 8).

While knowledge about electrophysiological dynamics of single neurons *in vivo* during the natural estrous cycle is needed, there is a considerable literature on pharmacological effects of hormone replacements studied in brain slice preparations.^41^ One prediction from such studies, combined with behavioral observations, is that the estrous phase will influence electrophysiological properties of neurons in many areas of the intact brain besides those that are part of the hypothalamic-pituitary-gonadal (HPG) axis. Here, we report on *in vivo* intracellular recordings, which show that the AOB mitral-tufted neurons are relatively hyperpolarized in estrus compared to in diestrus. Our results add to very few studies describing natural estrous cycle-dependent changes *in vivo* outside of the HPG axis, such as intrinsic electrical state variations in striatal medium spiny neurons^42^ and alterations in the ongoing activity of fast-spiking neurons in the rat barrel cortex.^5^ Moreover, in estrus mitral-tufted neurons specifically fire ongoing action potentials in HF bursts with ISIs of ≤ 10 msec. To the best of our knowledge there is no previous report of a switch to HF bursting of output neurons in an estrous cycle-dependent manner *in vivo*. Work in slices has indicated increased bursting (not in HF range) in proestrus compared to diestrus, in anteroventral periventricular neurons of the hypothalamus.^43^

Whether the hyperpolarization and/or the spontaneous HF bursting activity in estrus are dependent on external synaptic input that varies with the estrous cycle and/or are maintained by the AOB microcircuit itself in response to hormonal changes, is intriguing. The modest increase in spontaneous locus coeruleus activity in estrus could e.g., lead to an increased tonic inhibition of mitral-tufted neurons via norepinephrine-dependent activation of the GABAergic GCs, or at onset provide an external signal to the AOB microcircuit to initiate the estrous pattern of HF bursting that we have found. Increased tonic inhibition *per se* has been shown to increase the propensity of thalamic neurons to fire action potentials in bursts.^44^ Our data are compatible with a model in which both the hyperpolarization and the HF bursting in estrus are dependent on norepinephrine stimulation on to GCs as well as the interplay between GCs and mitral-tufted neurons via reciprocal synapses (Figure 8).

It is of course reasonable to assume that hormonal changes are directly or indirectly involved in the estrus-specific changes in electrophysiological properties of the AOB microcircuit. Yet, there is important research to be done to show how natural levels of circulating hormones, including steroid hormones, reflect levels in brain tissue.^45^ Local synthesis and modification of steroids in the brain should be considered ^46^*.* Recent literature on steroid hormone regulated neuronal activities, and genes implicated in regulation of bursting, provides many interesting candidates such as e.g., GABA_A_ receptor, metabotropic glutamate receptor, hyperpolarization-activated cyclic nucleotide-gated channels, and T-type voltage dependent calcium channels, which may serve as starting points for further mechanistic research.^46^^,^^47^^,^^48^^,^^49^

The estrus-specific activities of mitral-tufted neurons potentially influence VNO-pheromone information processing. In general, hyperpolarization can result in that stronger stimuli are required for neurons to reach spike threshold. The addition of an HF bursting pattern to a “default” diestrus type of spiking pattern, conceivably increases the possibility for the output neurons to relay more integrated information during estrus.^50^^,^^51^ Moreover, inhibition of mitral-tufted neurons in response to aVCS/coitus may be a reoccurring theme, suggested by our demonstration of immediate inhibition during aVCS and that there e.g., is an at least several hour long post-mating inhibition of the mitral-tufted neurons’ excitability as studied by *ex vivo* recordings.^52^

Brain *in vivo* responses to vagino-cervical stimulation are scarcely studied. By *in vivo* patch recordings we find that the developed aVCS protocol evokes a moderate stimulus-locked inhibition of the spontaneous ongoing spike output of mitral-tufted neurons, specifically in estrous females. We show that this spike inhibition coincides with a prolonged aVCS-driven activation of the inhibitory interneurons in the GC layer. This estrus-specific long GC activation could be due to an extended aVCS-driven presynaptic norepinephrine release from innervating locus coeruleus fibers, or slower postsynaptic adaptation to norepinephrine in GCs. Then again, we find that aVCS-driven spike inhibition in mitral-tufted neurons in estrus requires communication via the reciprocal synapses as blocking α1-adrenergic receptors as well as inhibiting communication by BAPTA infusion in mitral-tufted neurons, prevent the aVCS-evoked spike inhibitory response. The observation that both α1-adrenergic receptor block and intracellular BAPTA also reduce the spontaneous HF bursting of mitral-tufted neurons (discussed previously) is in line with the suggestion that HF bursting may facilitate the spike inhibitory response to aVCS, observed in estrus. Moreover, the effect of action potential backpropagation in dendrites on generating calcium transients in neurons is highest for bursts with ISIs of < 20 msec (i.e., including the HF bursting range) in AOB slices^53^ and measurement in the main olfactory bulb *in vivo* shows that inhibition via reciprocal synapses is more powerful when mitral neurons generate HF bursts.^20^^,^^54^ Thus, results from the BAPTA experiment allow us to suggest that the prolonged activation of GCs in estrus may depend on a more effective recruitment of reciprocal synapses, facilitated by the spontaneous HF bursting of the mitral-tufted neurons in estrus, rather than a prolonged activation of the locus coeruleus to GC synapse (Figure 8). A prolonged activation of reciprocal synapses will increase the duration of glutamate release from mitral-tufted neurons on to the GC part of the reciprocal synapse. The increased current could depolarize GC somas,^55^ which should be possible to readout as a prolonged LFP response in the CG layer.

One plausible addition to this scenario is that when the HF bursts back-propagate in mitral-tufted dendrites the resulting increased glutamate release in the reciprocal synapse, when co-incident with norepinephrine-evoked depolarization, may recruit NMDA receptors in GCs that in turn increase GABA release. NMDA receptors are involved in effective inhibition of main olfactory bulb mitral neurons by GCs *in vitro.*^55^^,^^56^ It is also possible that an increased glutamate release by mitral-tufted neurons might recruit slower perisynaptic^57^ metabotropic receptors on GCs which have been implicated in reciprocal synapse dependent inhibition *in vitro* in AOB.^18^^,^^58^

There is more information about *in vivo* AOB responses to VNO-pheromone stimulation. A multi-unit recording study in mice of mixed sexes demonstrates that AOB neuronal responses to VNO-pheromones even may disfavor temporal accuracy.^59^ A recent finding is that depression of responsiveness to the mating male’s pheromones is confined to the later part of the response.^60^ Taken together, the temporal characteristics of the response to VNO-pheromones is very different from the relatively stereotyped modulation of mitral-tufted neurons we find in response to aVCS, which is locked in time to the stimulus. As complex behavioral sequences, like mating, require timing information for behavior to be properly expressed, one suggestion is that somatosensory information produced during coitus could provide a time component as VNO-pheromonal information does not appear to provide time resolved information.

As discussed previously, spontaneous HF bursting may be central for the AOB microcircuit response during the few hours of estrus. A non-mutually exclusive possibility is that HF bursting may be relevant to AOB’s integration of sensory information about coitus and VNO-pheromones.^12^ Such integration is important for long term shaping of the mitral-tufted neuron’s activation pattern specifically in response to the mating male’s VNO-pheromones, so that the physiological memory trace is formed which leads to decreased endocrine response to his scent.^9^^,^^59^^,^^61^ A successful encoding of the mating male’s pheromones is needed to avoid that his individual pheromones later cause the classical pregnancy block. In other systems firing action potentials in bursts confers a higher degree of sensitivity to plasticity changes of synaptic inputs,^62^ reviewed in Zeldenrust et al..^63^

We show that the AOB microcircuit properties during estrus set the stage for coitus stimulation to inhibit the spike output neurons more effectively, possibly via HF bursting-dependent enhancement of the efficacy of reciprocal synapses. The results are not predictable from *in vitro* studies, conceivably due to the added complexity of input to AOB *in vivo* by ongoing brain activity. Our integrative physiological approach to address responses to coital stimuli is possible to extend to other brain areas that are directly or indirectly influenced by the female’s reproductive state.

### Limitations of the study

There is an overall lack of detailed literature on the afferent circuitry for vagino-cervical stimulation, including that to the locus coeruleus, in laboratory rodents. Recently data are pointing toward previously unknown partitionings of locus coeruleus [32] and gap junctions between pairs and small clusters of neurons,^64^ underscoring that more detailed knowledge on subnetworks within locus coeruleus is needed. Locus coeruleus releases norepinephrine in AOB upon mating, or aVCS, in the mouse [10, 14]. If the increased number of c-Fos positive locus coeruleus neurons we show in response to aVCS, are identical to those projecting to the AOB, is not specifically addressed in here, but the data show that the calibrated aVCS recruits locus coeruleus.

The effect of a α1-adrenergic receptor blocker on the aVCS-evoked local field responses was studied in estrous females. The similar amplitudes and latencies of the local field response in the mitral-tufted layer, together with the similar degree of activation of locus coeruleus neurons in estrus and diestrus, suggest that the responses are dependent on α1-adrenergic receptors in both stages of the estrous cycle. *In vivo* patch recordings of single neurons showed that spontaneous action potentials with ISIs in the HF bursting range (≤10 msec), are significantly more frequent during estrus and are also inhibited by the α1-adrenergic receptor blocker. Moreover, ISIs of e.g., 11–30 msec, which are common to estrous and diestrous females, were also prevented by this blocker, suggesting that the different spontaneous spiking patterns are also dependent on norepinephrine throughout the cycle. Blocking of α1-adrenergic receptors is not accompanied by a generalized suppression of spiking. These results suggest that the α1-adrenergic receptor has selective effects on the temporal firing structure of AOB output neurons.

The AOB has a laminar organization of somas and a spatial segregation of dendritic inputs, which are advantages when studying the functional complexity of an intact microcircuit *in vivo*. This *in vivo* study reveals microcircuit properties such as prolonged response to aVCS in GCs in estrus, which could serve as a starting point when employing innovative, optical approaches for functional dendritic calcium imaging approaches in deep brain structures *in vivo*.^65^ Such studies could be expected to generate new insight into dynamic synaptic mechanisms and the integration of synaptic information in dendrites, throughout the estrous cycle in AOB.

## STAR★Methods

### Key resources table

**Table:** 

REAGENT or RESOURCE	SOURCE	IDENTIFIER
**Antibodies**		

Polyclonal serum against c-Fos,	Synaptic Systems, Germany	RRID: AB_2231974
Primary chicken polyclonal against TH	Abcam, UK	RRID: AB_1524535
Secondary anti-chicken DyLightTM 594	Jackson Immunoresearch, UK	RRID: AB_2340373
Fluorescein–labelled donkey anti-rabbit IgG	Jackson Immunoresearch, UK	RRID: AB_2313584

**Chemicals, peptides, and recombinant proteins**		

Dexamethasone	Merck, Germany	D4902
Bupivacaine	Astrazeneca, Sweden	Vnr 169912
Thermal controller and rectal probe	WPI, US	ATC-2000
Paraformaldehyde in phosphate-buffered saline (PBS)	Carl Roth, Germany/Medicago Sweden	P087.1/09-8912-100
K gluconate	Sigma Aldrich	G4500
HEPES	Sigma Aldrich	H3375
Phosphocreatine Na	Sigma Aldrich	P7936
KCL	Sigma Aldrich	P9666
ATP Mg	Sigma Aldrich	A9187
GTP	Sigma Aldrich	G8877
BAPTA K salt	Molecular Probes	6806
Vaseline jelly	Aco, Sweden	260349
WB4101	Tocris, Bioscience, Uk	946
Fluoro-GoldTM	Fischer Scientific Fluorochrome, US	NC0560981
Antiseptic ointment	Dechra, UK	Fuciderm TM
Ketamine+xylazin i.p. solution	Pfeizer/Bayer	Vnr 150086/Vnr 023572
Sucrose	Fischer Scientific	S/8600/60
Normal donkey serum	Jackson immunoresearch, UK	017-000-001
Triton X-100	Sigma-Aldrich	X-100
Slice mounting medium	Vector Laboratories, US	Vectashield H-1200
DiI	Merck, Germany	468495

**Experimental models: Organisms/strains**		

Mouse C56B6, females	Charles River, Germany	Mouse C56B6J

**Software and algorithms**		

Patchmaster	HEKA, Germany	V2X73.1 2014
Spike 2	CED, UK	V 6,09
Matlab	Mathworks, US	V 2016b
Origin Pro	Origin lab, US	V 2019
Zen software	Zeiss, Germany	2,3 sp1 blue
Photoshop	Adobe, US	C2 version
Illustrator	Adobe, US	CS6 version
Neurolucida and Neuroexplorer softwares	MBF Bioscience, US	V 2016

**Other**		

Patch clamp Amplifier	HEKA, Germany	EPC800
Signal conditioners (filter and amplifier)	Npi elektronik, Germany	DPA-2FL
XYZ manipulator	Luigs und Neumann, Germany	SM-6
Iontophoretic device	Stoelting, US	Midgard 51595
Pressure injection device	Sigmann Elektronik, Germany	NIM module
Piezoelectric wafer	Pi-piezo technology, Germany	Shear plate
Vibratome	Campden Instr, UK	5100mz
Freezing microtome	Leica, Germany	SM2010R
3D printer	ZYYXlabs, Sweden	ZYYXpro printer
Apotome microscope	Zeiss, Germany	Axio Imager M2 with CMOS Camera (Hamamatsu, Japan)
Universal electrode puller	Zeitz, Germany	DMZ
Vertical pipette puller	Narishige, Germany	PC-100
Borosilcate glass pipette, 1.5 outer diameter, with filament	Hilgenberg, Germany	Code 1810025
Olympus microscope	Olympus, Japan	CX31
100x air objective	Olympus, Japan	LMPlanFL 100x/0.8

### Resource availability

#### Lead contact

Further information and requests for resources and reagents should be directed to and will be fulfilled by the lead contact, Paolo Medini (paolo.medini@umu.se).

#### Materials availability

This study did not generate new unique reagents.

### Experimental model and subject details

#### Animals, husbandry and estrous status

Adult sexually naïve C57BL/6J female mice (postnatal day 90 to 120, 20-25 g body weight; (Charles River, Germany) were housed in a dedicated room without males, at the Umeå center for comparative biology with *ad libitum* access to food and water. The mice were on a reversed 12:12 h light/dark cycle so that experiments were performed during the animals’ active phase at night. All animal experiments were approved by the local ethics committee for animal research at the Court of appeal for the upper Northern region of Norrland (Umeå, Sweden) with approval number A26-2018. The committees in Sweden are chaired by two lawyers. Additional members are six researchers/animal care takers, four laypersons suggested by local political parties and two laypersons from animal welfare organizations.

In the morning before start experiment (around 9 am) the vagina was flushed with 50 μL of physiological 0.9% NaCl and the sample was observed under transmitted light, bright-field microscopy. Phase of the estrous cycle was scored based on the cytology of the epithelial cells.^66^ Animals were excluded in case of irregular cycling or if the phase was ambiguous. Females in estrus or diestrus were included in experiments.

### Method details

#### Surgical procedures

Anesthesia was induced by isoflurane gas (Piramal, UK). Anesthetic depth was maintained using around 1.5% isoflurane and the concentration was increased to 2.5-3.0% during surgery. Anesthetic depth was monitored by assessing the withdrawal and corneal reflexes as well as pulse and breathing rates.^67^^,^^68^ Ophthalmic ointment was used (Alcon, USA). Before start of surgery, 0.01 mgxkg^−1^ dexamethasone (Merck, Germany) was injected i.p. to prevent cerebral cortical and mucosal edema. Local anesthetic (bupivacaine 2.5 mgxmL^−1^; Merck, Germany) was applied topically to the area of incision. Animals were held in a custom-made stereotaxic apparatus. Body temperature was maintained at 37°C during surgery and recorded by a feedback-looped thermal controller (WPI, USA) by a miniaturized thermocoupled rectal probe. Electrocardiogram and breathing rate were monitored via a small dedicated amplifier and a custom-made plathysmometer around the animal’s abdomen. Animal condition was monitored during the experiment by measuring heart and breathing rate, minimal but detectable pinch and corneal reflexes, as well as by clinical assessment of paw colour as indicator of peripheral perfusion. Unfavorable development of these signs is suggestive of deterioration of the animal, typically preventing stable extra- or intra-cellular recordings and such animals were excluded. A craniotomy (1 mm in diameter) over the prefrontal cortex, centered 1.0 mm posterior to the inferior cerebral vein and 1.0 mm lateral from the midline allowed for extracellular LFP or *in vivo* whole-cell recordings. After electrophysiological recordings, animals were sacrificed with an overdose of isoflurane and subjected to transcardial perfusion with 4% paraformaldehyde in phosphate-buffered saline (PBS). After overnight post-fixation of the dissected brain in the same fixative, 100 μm thick sagittal sections were cut with a vibratome (Campden Instruments, UK). When the pipette tip was painted with DiI in order to reveal positioning of the electrode, then the sections were mounted immediately. Alternate sections were processed for further histological analysis.

#### Artificial vagino-cervical stimulation

For aVCS, a custom-made 3D printed vagino-cervical stimulator (ZYYXpro printer, Sweden) was mounted on a piezoelectric membrane (PI-Piezo technology, Germany; Figure S1A). The vagino-cervical stimulator was lubricated with Vaseline jelly (Aco, Sweden) and manually inserted until touching the cervix in the fully anaesthetized mouse. Thus, mice were not conscious of the aVCS stimulus and experiments were terminal right after discontinuation of the stimulation. Correct placement of the stimulator in relation to the cervix surface was verified post-mortem (N=5 mice, Figure S1C). The displacement of the vagino-cervical stimulator was calibrated (Figure S1B). aVCS lasted 5 sec (10 Hz stimulation), followed by an interstimulus interval of 15 sec. This 20 sec stimulus was repeated 30 times, for a duration of the entire aVCS protocol of 10 min (Figure S1D).

#### Electrophysiology 1: local field potential recordings

##### Data acquisition

Borosilicate glass pipettes (0.8–1.2 MΩ) filled with 0.9% NaCl were inserted into the prefrontal cortex at a 60 degrees angle from the vertical axis until the AOB was reached. A ground wire was positioned on the skull bath made with acrylic cement. The signal was band-filtered (0.1–100 Hz) and amplified (1000X) with a differential amplifier (npi, Germany). The signal was then digitized (1 kHz) and acquired through an analog/digital board (CED, UK) and pre-analyzed via Spike2 analysis software (CED, UK). Stimuli (30-40 V amplitude applied to the piezoelectric device) causing saturating responses were presented at least 30 times and responses were trigger-averaged (Figure S1D). At the end of the recording, the pipette was painted with DiI (Invitrogen-ThermoScientific, USA) while the mouse was still in the stereotaxic frame and the pipette was reinserted at the recording depth for subsequent histological examination for localization (Figures 2A and 3B).

##### Data analysis

Data analysis was performed using custom-written routines in MATLAB (Mathworks, USA) or software Spike2 (CED, UK). The raw signal was further low pass filtered using a second-order Butterworth filter at ≤25 Hz. For grand average calculations traces were aligned to the mean value of the baseline, representing a 5 sec pre-stimulus period (Figure S1D right). The peak amplitude relative to baseline and time to peak were measured within the first 2 sec from stimulus onset, with the exception of LFPs recorded from the GC layer for which two time windows were defined and analyzed; 0-2 and 2-5 sec after stimulus onset (yellow and blue areas in Figure 3). For comparison of LFP amplitudes from the GC layer in estrus and diestrus, normalization of the signal negative peak of each recording was applied (peak made equal to 1, Figure 3A).

#### Electrophysiology 2: Whole-cell patch-clamp recording

##### Data acquisition

*In vivo* whole-cell recordings were obtained using a blind approach technique.^36^ Patch pipettes (5–8 MΩ) were filled with intracellular solution (in mM: 135 K-gluconate, 10 HEPES, 10 phosphocreatine-Na, 4 KCl, 4 ATP-Mg, 0.3 GTP; pH 7.2, 291 mOsm). Pipettes were generated by a 1.5 mm (outer diameter) filamented borosilicate glass pipettes (Hilgenberg, Germany, code 1810025) and were pulled to 6-9 MΩ pipettes with a horizontal or vertical puller (DMZ Universal Puller from Zeitz, Germany, or PC-100 puller, Narishige, Japan). Positive pressure (300–400 mbar) was applied while lowering the pipette into the brain and controlled by a manual seal sucker pressure controller (Sigmann elektronik, Germany). Pipette tips and shape (internally concave, tip diameter ca 1 μm) were controlled with an Olympus CX31 microscope equipped with a 100x air, long working distance objective (Olympus LMPlanFL 100x/0.80). Once the start of the depth range of interest was reached, the pressure was lowered to 30 mbar. Cells were then searched in voltage clamp modality while advancing in 1-2 μm steps along the pipette axis. When cells were approached, the pressure was relieved to zero*,* the pipette potential was hyperpolarized, and a light suction was applied to facilitate gigaseal formation. After capacitance compensation, typically a ramp of negative pressure led to whole-cell configuration. Recordings were performed with using an EPC800 amplifier (HEKA, Germany) in current clamp mode. The signal was digitized at 20 kHz and acquired using Patchmaster software (HEKA, Germany). Seal resistance was higher than 2 GΩ. Spike height and overall V_m_ were stable throughout recordings in the recorded neurons included in this study. Series resistance ranged between 50-140 MΩ. The nominal depth readings of whole-cell recordings were determined relative to the contact point with the pia surface (of prefrontal cortex) from the z-axis readout of the micromanipulator (Luigs & Neumann, Germany). Recording duration ranged from 30 to 90 min. A maximum of two whole cell recordings were performed per animal. Stimuli were presented at least 30 times as during extracellular recordings.

For 1,2-bis(o-aminophenoxy)ethane-N,N,N′,N′-tetraacetic acid (BAPTA)-K experiments (Figure 6), patch pipettes were filled with a 20 mM BAPTA intracellular solution (see above, with the exception that K-gluconate was reduced to 115 mM). A 150 pA depolarizing pulse (500 msec duration) was applied on a tonic current injection of 150 pA, to ensure that neurons fired action potentials reliably during the test pulse (Figure 6A bottom).

##### Data analysis

Patch clamp data were analyzed using custom-made routines in MATLAB.

###### Supra-threshold (action potential, spike) activity

Action potentials were identified based on peak values above a manually set voltage threshold with decrease in V_m_ within 2 msec before and 3 msec after the peak while reaching the action potential threshold value (the latter within the first time frame and defined as the point of maximal acceleration of the V_m_^69^). To compute peristimulus time histograms of action potential counts, 100 msec binning was applied. ISIs from all neurons within a given experimental groups were binned at 10 msec for the cumulative plots and at 1 msec for the high resolution histograms (Figures 4C, 6F and 7D).

###### Sub-threshold (synaptic) activity

For analysis of the sub-threshold activity, action potentials were removed by linear interpolation and sweeps were averaged. aVCS-driven changes in membrane polarization were then measured by comparing averaged V_m_ before and during aVCS.

*V*_*m*_*moving variance analysis* (Figure 5E): was calculated for individual sweeps. First the local minimum of action potential firing during stimulation was established and data for a time window of ±0.5 sec around this time point was analyzed. The local minimum of action potential firing was calculated on the filtered peristimulus time histogram by Gaussian zero-phase forward and reverse digital IIR filter (8 samples window in peristimulus time histogram).

*Additional V*_*m*_*analysis* (Figures 4E, 6G, and 7E)*:* To measure the time spent at various V_m_ for each experiment, the voltage values for all sweeps were grouped into bins from -80 mV to -20 mV with 0.25 mV steps. Bin values were normalized by total number of samples across all sweeps and multiplied by 100 to get values in percentage. The cumulative distribution obtained for each neuron was averaged among neurons according to experimental condition (estrus, diestrus or pharmacological treatment). Median V_m_ values were measured from such plots.

#### *In vivo* pharmacology

To antagonize the α1-adrenergic receptors extracellularly during both extracellular and intracellular recordings (Figures 2G and 7), borosilicate pipettes used for LFP recordings (see above) were filled with WB4101 (2-(2,6-Dimethoxyphenoxyethyl)aminomethyl-1,4-benzodioxane hydrochloride; Tocris Bioscience, UK) dissolved in 0.9% NaCl to a concentration of 30 μM.^16^ In order to avoid leak of inhibitor during LFP baseline pre-recording, gentle negative pressure was applied during the pre-drug application phase. Then gentle positive pressure was applied with a pressure-injection device (NIM module, Heidelberg, Germany) until 0.5 mL of WB4101 solution or vehicle was infused over 5 min. The injection was the same for both extra- and intra-cellular recordings. Recordings were then repeated 30 min after drug or vehicle control injection (excluding perfusion time).

#### Histology

##### Fluoro-Gold injections in AOB

*In vivo* iontophoretic placements of a small deposit of 1% solution of retrograde tracer Fluoro-Gold™ (Fluorochrome, USA) were done unilaterally in the AOB using borosilicate glass pipettes (tip diameter 15-20 μm). The surgical and stereotaxic approach was the same as for electrophysiological recordings. Injections were made using positive squared pulses of current (5 μA of amplitude) for 15 min (7 sec on/7 sec off) via a current source (Stoelting, USA). The pipette was left in place after injection for an additional 10 min for complete diffusion. After surgery antiseptic ointment (Fuciderm™, Dechra, UK) was applied to the incision site and the animals were individually housed in ventilated cages for recovery. After 10 days, the animals were deeply anaesthetized with ketamine (90mg/kg) and xylazine (10mg/kg) solution (0.01 ml/g i.p.) and thereafter perfused transcardially with 4% paraformaldehyde in PBS, the brains collected and post-fixed for 2 hr before transfer to sucrose solution (30% in PBS) at 4°C for cryoprotection (Figures 1A and 1B).

##### Histology and immunofluorescence of locus coeruleus

###### Fluoro-Gold detection in locus coeruleus

60 μm thick sagittal brain sections were cut with a freezing microtome (Leica, Germany) and sections collected in PBS. The Fluoro-Gold injection site was confirmed using a fluorescence microscope before further histological processing. Serial sections were analyzed for tyrosine hydroxylase to delineate the borders of locus coeruleus (Figures 1B and 1C). Sections were incubated in blocking solution containing 3% normal donkey serum (Jackson Immunoresearch, UK) and 0.2% Triton X-100 in PBS for 1 hr followed by incubation in blocking solution with 1% normal serum and primary chicken polyclonal antibodies against tyrosine hydroxylase (1:1000; #76442; Abcam, UK) at 4°C for 72 hr. Specific immunoreactivity was revealed after incubation with secondary DyLight™ 594 antibody (1:250; #703-475-155; Jackson Immunoresearch, UK) in 0.2% Triton X-100 in PBS for 90 min. Sections were mounted using Vectashield mounting media (Vector Laboratories, USA).

##### Vagino-cervical stimulation and c-Fos staining in locus coeruleus

Quantification of c-Fos positive cells in locus coeruleus was done for four groups of mice (N=5 each; Figures 1C–1E). Analysis was done for females in estrus and diestrus. The stimulated groups underwent a single aVCS (protocol as for electrophysiology, excluding surgical preparation). Control groups were sedated and handled identically, but without inserting the stimulator. Brains were directly processed for further analysis as outlined above. Serial 60 μm sagittal sections were stained for tyrosine hydroxylase as above, and in addition with a polyclonal antiserum against c-Fos (1:5000; #226003; Synaptic Systems, Germany). For detection of c-Fos fluorescein-labeled donkey anti-rabbit IgG (1:250; Jackson Immunoresearch, UK) was used.

#### Image acquisition and quantification

Transmitted light, bright-field images were acquired on an Axio Imager M2 microscope (ZEISS, Germany) with CCD device (Axiocam 503, ZEISS, Germany). Fluorescent images were acquired with the same microscope, but using a CMOS (ORCA-Flash 4.0 LT) camera (Hamamatsu, Japan). A program employing Zen software (blue edition) was used for both type of image acquisitions (ZEISS, Germany).

Filter-blocks used were: 1) a wide band ultraviolet excitation filter (exc. 323 nm, em. 620 nm) for Fluoro-Gold; 2) Zeiss filter set 64HE (exc. 587/25 nm, em. 647/70 nm) for tyrosine hydroxylase; 3) Zeiss filter set 38 (exc. 470/40 nm; em. 525/50 nm) for c-Fos. The high magnification images (Plan-Apochromat 20X objective) were obtained using the ApoTome.2 module (Figure 1C). The tridimensional reconstruction of locus coeruleus was based on 4-5 serial sections per animal with Neurolucida 360 software (MBF Bioscience, USA). c-Fos positive cells within the borders of locus coeruleus were manually marked and automatically quantified by Neurolucida explorer (MBF Bioscience, USA; Figure 1D). Linear corrections were made for brightness and contrast, and the same corrections were applied for all sections in parallel, using Photoshop software (version CS2 Adobe, USA). Locus coeruleus borders were determined by using both the mouse brain atlas^70^ as well as the spatial extent of the tyrosine hydroxylase immunofluorescence signal as reference (Figure 1E).

### Quantification and statistical analysis

Statistical analyses were performed using OriginPro (OriginLab, USA) or MATLAB1. Normality was controlled with Shapiro-Wilk test. For normally distributed data, mean±SEM was computed and otherwise medians (+ Q1 and Q3) were calculated. Normally distributed data were compared using either paired or unpaired t-test whereas non-normally distributed data were compared employing MWU-test or WSR-test statistics. When indicated, interactions between estrus state and physiological parameters were done with 2-way ANOVA or with a mixed-model design ANOVA, depending on whether the parameter measurement was unpaired or paired, respectively. The K-S-test was used to compare cumulative distributions. For all comparisons, the threshold for statistical significance was set at alpha=0.05. Red lines in paired dot plots indicate mean or medians depending on whether data were normally or non-normally distributed.

### Additional resources

Further information and requests for resources and reagents should be directed to and will be fulfilled by the lead contact, Paolo Medini (paolo.medini@umu.se).

## Data Availability

• Data reported in this paper will be shared by the lead contact upon reasonable request. • This paper does not report original code. • Additional information required to re-analyse the data reported in this paper is available from the lead contact upon reasonable request.
