# Supplementary material for: In vivo spontaneous activity and coital-evoked inhibition of mouse accessory olfactory bulb output neurons
Source: iScience. 2023 Aug 7;26(9):107545. doi: 10.1016/j.isci.2023.107545 (PMC10470370; doi:10.1016/j.isci.2023.107545)
Supplement: Document S1. Figures S1–S3 [file mmc1.pdf]

iScience, Volume *26*

Supplemental information

In vivo spontaneous activity and coital-evoked inhibition of mouse accessory olfactory bulb output neurons

Paolo Lorenzon, Kamil Antos, Anushree Tripathi, Viktoria Vedin, Anna Berghard, and Paolo Medini

SUPPLEMENTARY FIGURES AND FIGURE LEGENDS

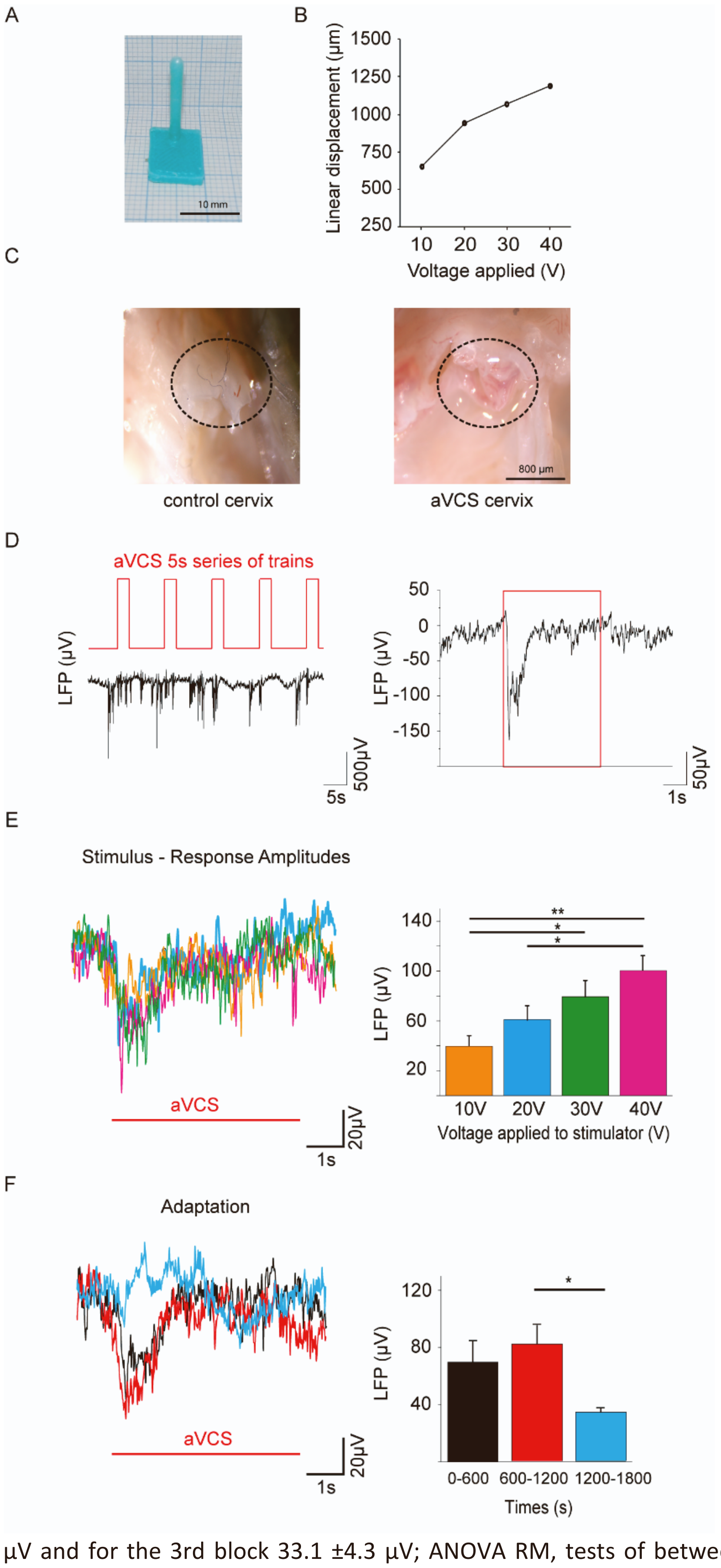

Figure S1. Calibrated artificial vagino-cervical stimulation protocol and evoked potentials in the accessory olfactory bulb. (A) Shows 3D printed "dildo-like" stimulator used for artificial vagino-cervical stimulation (aVCS). The stimulator was glued to a piezoelectric actuator. (B) Relationship between the displacement of the stimulator and voltage applied to it. (C) Post-mortem examination of vagina and cervix from an unstimulated and stimulated female. There is slight stimulation-induced hyperemia of the cervical tissue (encircled). (D) Left: example of local field potential (LFP) raw trace during aVCS (black). LFP was recorded in the AOB at a depth corresponding to the mitral-tufted layer. Right: example of aVCS-EP. (E) Left: example of aVCS-EP responses to different aVCS input stimulation amplitudes. Right: aVCS-EP peak amplitude increases with stimulation (N=4 estrus females). At 650µm displacement, the average amplitude (±SEM) of the response was 39.8±3.4 µV; at 950µm 59.9±13.2 µV; at 1050µm 79.4±13.0 µV and at 1200µm 103.3±10.2 µV. Responses to stronger stimuli demonstrated statistically significantly larger amplitudes (ANOVA RM, tests of between subjects effects: p=5.7E-4; followed by Fisher post-hoc test). At 650-1050µm: p=0.035; 650-1200µm: p=0.003; 950-1200µm: p=0.023 (N=4; ANOVA-RM, p=5.7E-4, Fisher post-hoc test, *p<0.05, **p<0.005).

(F) Test of long-term adaptation of aVCS-EPs is shown. Left: examples of aVCS-EP responses upon repetitive stimulus presentation (0-600 sec, black trace; 600-900 sec, red trace; 900-1200 sec, blue trace). Right: shows a drop in responsiveness from the second to third block, which suggests that the response is robust for a prolonged time, before adaptation can be observed (N=5; average response amplitudes for 1st block 70.7±14.0µV; for the 2nd 82.0±14.5 µV and for the 3rd block 33.1 ±4.3 µV; ANOVA RM, tests of between-subjects effects: p=2.3E-4: followed by Fisher´s post-hoc tests (1st-2nd block: p=0.565; 2nd-3rd block: p= 0.031; 1st-3rd block: p=0.079) *p<0.05). RELATED TO MAIN FIGURE 2.

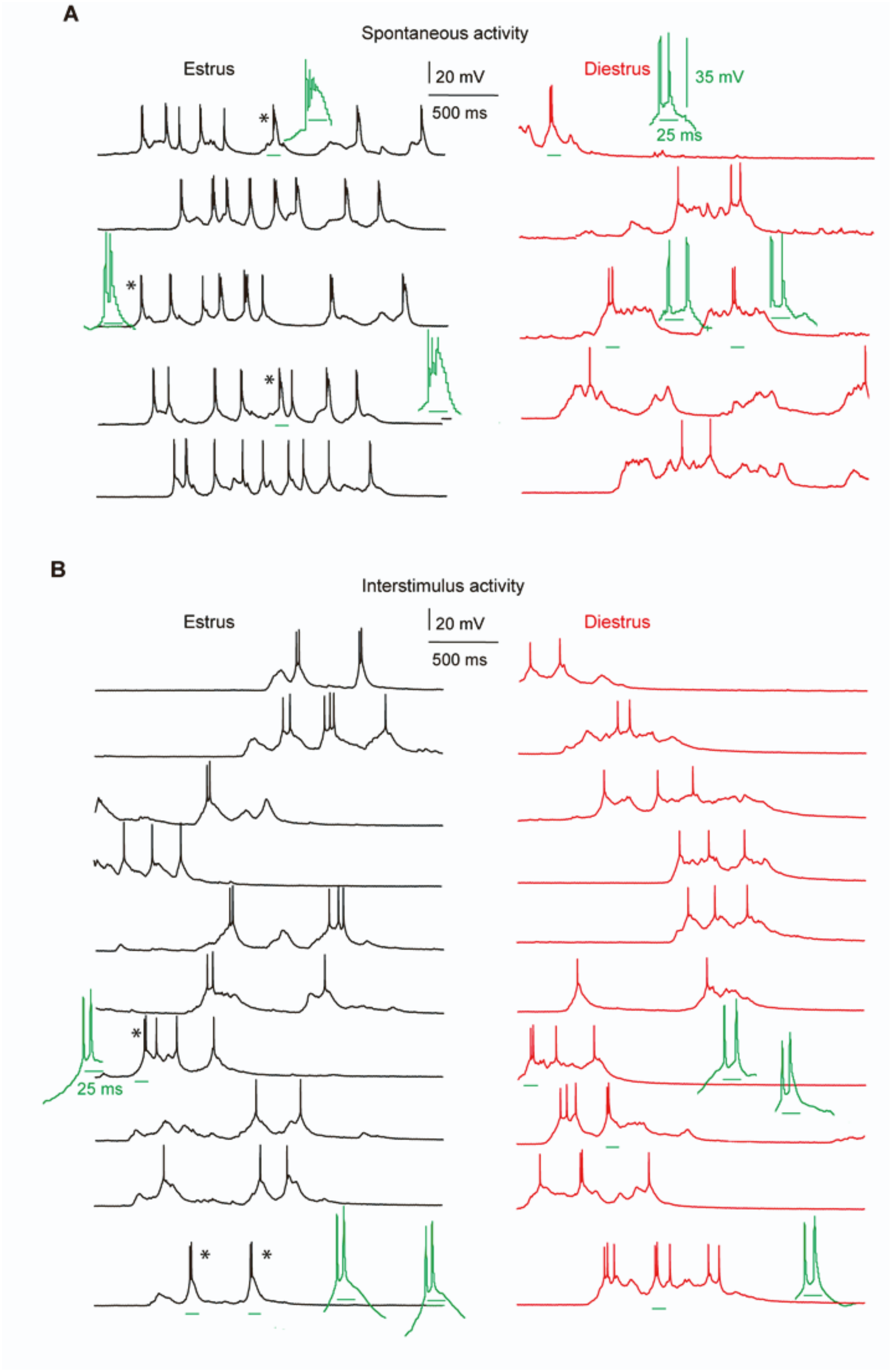

Figure S2. Time resolved images of individual spontaneous spikes, in estrus and diestrus. Traces from single mitral-tufted neurons, not stimulated by aVCS. Traces in (A) are from neurons that have never experienced aVCS (like in **Figure 4**), while those in (B) are from non-stimulated periods between aVCS periods (like in **Figure 5**). Single spikes close in time have been magnified further and are shown in green. * Denotes when two spikes are ≤10 msec apart. Such short spike intervals are quite rare during estrus and significantly more rare during diestrus. For quantificaton please see **Figure 4C**. RELATED TO MAIN FIGURES 4 AND 5.

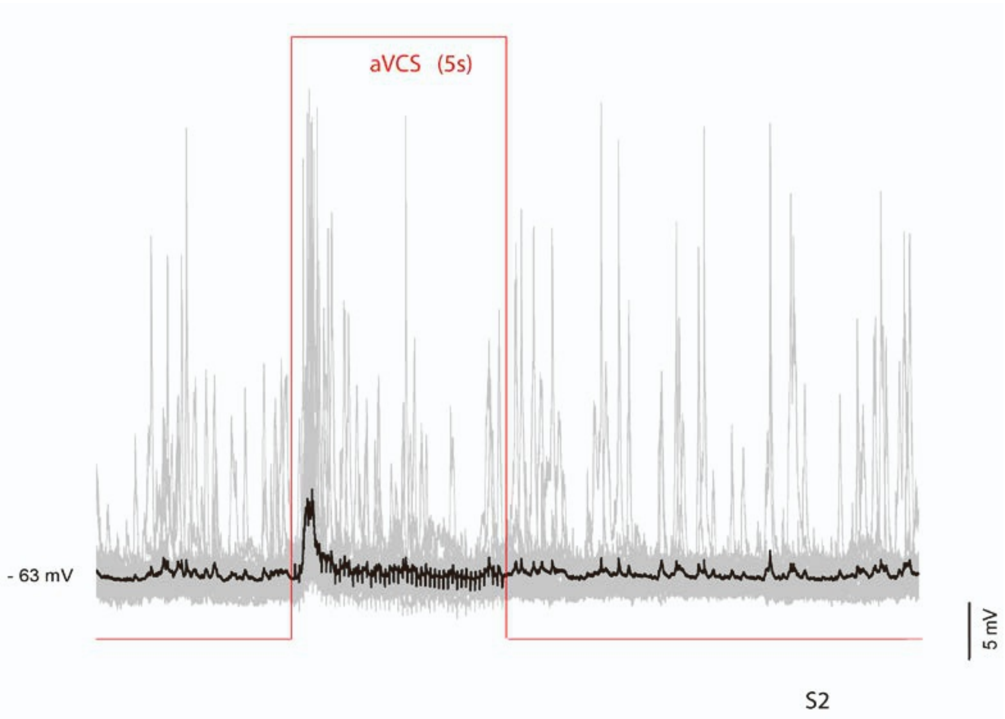

Figure S3. A minor fraction of neurons in the mitral-tufted layer with early excitatory postsynaptic potentials. Example of a neuron displaying an EPSP during the first sec of aVCS. The fraction of cells with this behavior was tentatively independent of the estrous cycle as 2 such neurons were found in estrus, 3 in diestrus and 1 in the BAPTA group (none in the WB4101 group). The inclusion criterium was a depolarization of at least 3 mV within the first second of aVCS in the averaged subthreshold trace (black; gray is the overlay of all the recorded sweeps). The time kinetics of such intracellular EPSPs were similar to that of the aVCS-EP in the mitral-tufted layer. This cellular response could originate from i) signal integration within the microcircuit, ii) cells representing a small fraction expressing the α1D-adrenergic receptor isoform [1] or iii) the so called sparse external granule cells placed in the mitral-tufted cell layer [2]. RELATED TO MAIN FIGURE 5.

1. https://mouse.brain-map.org/experiment/show/69236807

2. Zhang, X., and Meeks, J.P. (2020). Paradoxically Sparse Chemosensory Tuning in Broadly Integrating External Granule Cells in the Mouse Accessory Olfactory Bulb. J Neurosci *40*, 5247-5263.
